# Molecular dynamics guided identification of a brighter variant of superfolder Green Fluorescent Protein with increased photobleaching resistance

**DOI:** 10.1038/s42004-025-01573-4

**Published:** 2025-06-05

**Authors:** Rochelle D. Ahmed, W. David Jamieson, Danoo Vitsupakorn, Athena Zitti, Kai A. Pawson, Oliver K. Castell, Peter D. Watson, D. Dafydd Jones

**Affiliations:** 1 Molecular Bioscience Division, School of Biosciences, Cardiff University, Cardiff, UK; 2 School of Pharmacy and Pharmaceutical Sciences, Cardiff University, Cardiff, UK

**Keywords:** Protein design, Biophysical chemistry, Computational chemistry, Fluorescent probes

## Abstract

Fluorescent proteins (FPs) are a crucial tool for cell imaging, but with developments in fluorescence microscopy and researcher requirements there is still a need to develop brighter versions that remain fluorescent for longer. Using short time-scale molecular dynamics-based modelling to predict changes in local chromophore interaction networks and solvation, we constructed an *Aequorea victoria* GFP (avGFP) variant called YuzuFP that is 1.5 times brighter than the starting superfolding variant (sfGFP) with a near 3-fold increased resistance to photobleaching in situ. YuzuFP contained a single mutation that replaces the chromophore interacting residue H148 with a serine. Longer time scale molecular dynamics revealed the likely mechanism of action: S148 forms more persistent H-bond with the chromophore phenolate group and increases the residency time of an important water molecule. As demonstrated by live cell imaging, YuzuFP not only offers a timely upgrade as a useful green-yellow avGFP for cell imaging applications over longer time scales, but it also provides a basic scaffold for future avGFP engineering efforts.

## Abstract (web summary)

Fluorescent proteins are essential for cell imaging, yet brighter and more photostable variants are needed to meet advancing microscopy demands. Here, the authors use molecular dynamics modeling to develop YuzuFP, a GFP variant with enhanced brightness and photostability, offering a potential tool for extended live cell imaging.

## Introduction

Fluorescent proteins (FPs) are an indispensable tool in modern cell biology^1–4^, with protein engineering playing a pivotal role in generating useful variants from a relatively small pool of non-optimal natural starting points. However, there is still a continuing need to generate new FPs to match the improvements and requirements in cell imaging. Such parameters include improved brightness, resistance to photobleaching, longer “on” times (cumulative time spent in a bright state prior to photobleaching) and longer fluorescence lifetimes amongst others^5,6^. The original green fluorescent protein (GFP) from *Aequorea victoria* (avGFP) was, and still is, the underlying basic protein scaffold by which many FPs spanning the blue to yellow region are engineered ^7–10^. One of the first truly useful engineered versions of GFP still widely used today is EGFP (enhanced GFP)^11,12^. Modifying GFP to EGFP resulted in a shift in the dominant ground state chromophore from a neutral protonated form (CroOH; λ_max_ 395 nm) to the deprotonated charged phenolate form (CroO^-^; λ_max_ ~ 485 nm) with more favourable excitation and fluorescence properties. Since then, many derivatives of avGFP have been engineered (see FPBase^13^ www.fpbase.org/protein/avgfp/), including superfolder GFP (sfGFP)^14^. While sfGFP folds and matures quicker, and is very stable, key fluorescence properties such as brightness and photobleaching resistance are similar to EGFP (Table 1)^15^. Brightness can be improved for green-yellow versions of avGFP (e.g. Venus^9^ and Clover^16^) and related FPs (e.g. mNeonGreen^17^) but results in reduced resistance to photobleaching (Table 1)^15^.

**Table 1:** Spectral properties of sfGFP-H148X variants and other fluorescent proteins

FP	λ_max_ (nm)	ε (mM^−1^cm^−1^)	λ_EM_ (nm)	QY	Brightness (mM^−1^cm^−1^)	pKa	Relative photobleaching resistance
YuzuFP (sfGFP^H148S^)	492	61.4	511	0.92	56.5	6.2	1.8 (in vitro)^a^ 2.8 (cells)^a^
sfGFP^a,b^	485	49.0	509	0.72	35.3	6.1	1^a,d^
sfGFP^H148A^	398 497	36.5 15.2	511	0.35 0.54	12.8 8.2	N/D^e^	N/D^e^
EGFP^c^	488	56	508	0.67	37.5	6.1	0.81^d^
mNeonGreen^c^	506	113	517	0.80	90.4	5.7	0.72^d^
mClover^c^	505	105	516	0.84	88.2	5.9	0.27^d^
mVenus^c^	515	127	528	0.67	85.1	6.0	0.13^d^
mCherry^c^	587	85	610	0.30	25.5	4.5	1.41^d^

^a^Determined in this study.

^b^From Reddington et al.^31^.

^c^From the Cranfill et al.^15^.

^d^Based on t (1/2) values published by Cranfill et al.^15^. Values relative to sfGFP, which is set to 1.

^e^Not determined.

**Fig. 1:**
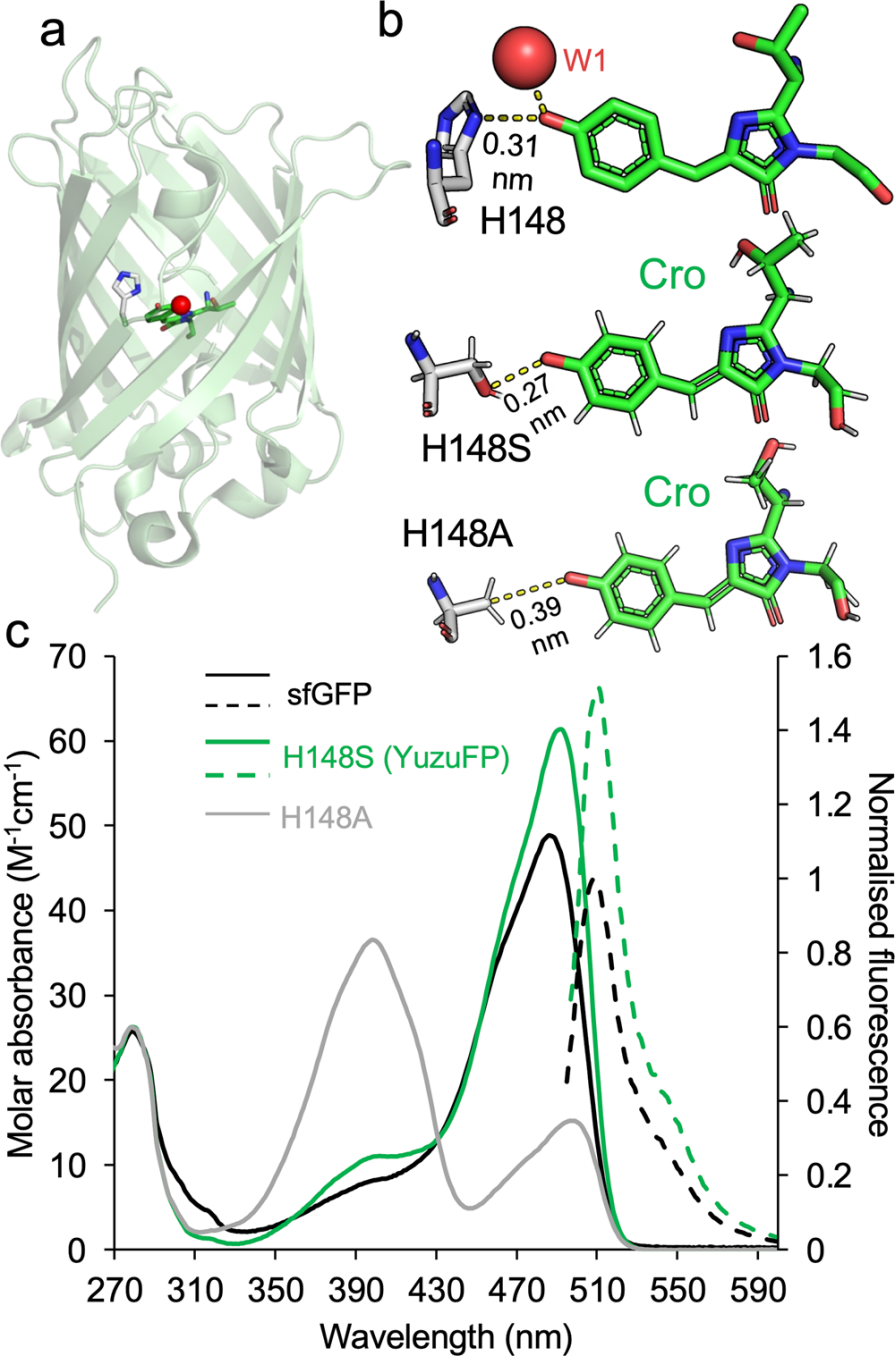
Engineering the interactions between residue 148 and the chromophore in sfGFP. **a** Overall crystal structure of sfGFP (PDB 2b3p) ^14^ with the arrangement of the chromophore (green sticks) relative to H148 (grey sticks) shown. **b** Comparison of the H148 interaction from the crystal structure of sfGFP and the short time scale molecular dynamics models of the H148S and H148A variants. Dashed yellow lines represent distances between Cro phenolate O and nearby heavy atoms. Other H148X models can be found in Supplementary Fig. 3. **c** Spectral characteristics of sfGFP (black), our newly designed H148S (also known as YuzuFP) (green) and H148A (grey). Solid lines represent absorbance spectra and dashed line emission spectra. Emission spectra for sfGFP and YuzuFP were recorded on excitation at 485 nm and 492 nm, respectively. Emission spectra were normalised to the sfGFP emission maximum. Source data for plots in (**c**) are provided in Supplementary Data 1.

One of the key determinants of avGFP’s fluorescence properties is the histidine at position 148. Together with a structurally conserved water molecule (here termed W1), H148 directly interacts with the chromophore phenolate group and is thought to play a key role in promoting and stabilising CroO^-^ in the ground state^18–24^ (Fig. 1a)^18,21,22,25^. H148 is also thought to be dynamic and has been observed to sample an alternate “open” conformation distant from the Cro^18,21,24,26^. In many non-avGFPs, histidine is replaced with a non-ionisable polar residue^27^ (Supplementary Fig. 1). For example, Dronpa^28^ has a high quantum yield (0.85) with S142 acting as the equivalent of H148; mCherry (as with many DsRed derived FPs) also has a serine at the position equivalent to H148. The recently developed high brightness and photobleaching resistant StayGold FPs^29,30^ have an asparagine (N137). Thus, H148 seems to be an uncommon choice in natural FPs for the crucial role played by this residue.

Here we assess how replacing the imidazole side chain of H148 in sfGFP modulates the charged state on the chromophore between CroOH and CroO^-^; replacement with a hydroxyl group of serine results in a brighter protein due to improved molar absorbance and quantum yield (QY). It also has improved resistance to photobleaching. Molecular dynamics simulations reveal the importance of more persistent H-bonding between residue 148 and the chromophore, and longer water residency providing insights into the molecular mechanism and leading to possible routes forward in how to engineer FPs for improved fluorescence properties.

## Results and discussion

### Importance of residue 148

The role played by H148 in promoting CroO^-^ in avGFP-derived proteins is well established^18,21–23,31–33^. However, H148 is not considered to play a critical role in sfGFP folding or chromophore maturation as mutants of H148 still fold correctly to generate a fluorescent protein^23,31,32,34,35^. H148 is thought to be dynamic^18,21,26^ and capable of occupying different conformations some of which break the interaction with the chromophore^24,31,36,37^. The most commonly observed form in crystal structures is shown in Fig. 1a but the configuration is non-optimal in terms of the plane of interaction (~140°) and the distance (0.31–0.35 nm) between the imidazole side chain and the chromophore (Supplementary Fig. 2). Even over short-time scale (10 ns) simulations the H-bond between the chromophore phenolate group and H148 is present for less than 5% of the time (Supplementary Table 1) and the structurally conserved nearby water molecule, W1 (Fig. 1b), has a low residency time (Supplementary Table 2).

The question then arises as to the importance of H148 and its imidazole side chain to avGFP fluorescence: Is histidine the optimal residue? Mutant structure modelling using approaches such as AlphaFold^38^ and Rosetta^39^ is hindered for FPs by their inability to incorporate the chromophore, and crucially for FPs, the local solvation environment. Furthermore, such static modelling does not consider structural relaxation and dynamic flux. Therefore, we initially undertook molecular modelling involving short time scale molecular dynamics (10 ns) to sample the 19 main canonical amino acids in place of H148 (Fig. 1b and Supplementary Fig. 3). Modelling suggests that apart from H148, only serine and asparagine retain a side chain configuration capable of making a H-bond with the Cro phenolate oxygen. The H148S model suggests the serine hydroxyl group forms a H-bond with the Cro phenolate oxygen that is shorter in distance (0.27 nm for the O-to-O distance (Fig. 1b and Supplementary Fig. 2) and the atomic configuration being close to planar (Supplementary Fig. 2). H-bond analysis also suggests the chromophore phenolate O forms a H-bond with S148 more frequently and even forms two H-bonds occasionally (backbone amine group as well as the side chain hydroxyl group) (Supplementary Table 1). Longer distances to the phenolate oxygen group and different relative side chain orientations are observed for H148N (Supplementary Fig. 3). Starting models of H148N before MD (thus no hydrogen present) suggests that either the amine or carbonyl can form the polar interaction depending on carboxamide rotation. However, after 10 ns of MD where the phenolate form of the chromophore is sampled only the amine forms a H-bond (Supplementary Fig. 3 and Supplementary Table 1). In contrast, the H148T mutation does not appear to form a persistent H-bond via the side-chain hydroxyl. Replacement of H148 with alanine retains the β-carbon facing towards the chromophore but removes any side chain H-bond potential to the chromophore (Fig. 1b and Supplementary Table 1). In the sfGFP H148C model, the cysteine side chain flips out likely due to the larger atomic radius of sulphur and the weaker Lewis base character of the thiol group compared to a hydroxyl group (Supplementary Fig. 3 and Supplementary Table 1). Models of the other mutations indicate no H-bonds with the chromophore phenolate will form (Supplementary Fig. 3).

We also considered H-bond frequency of the structurally conserved water molecule W1 (highlighted in Fig. 1b) over the 10 ns simulation. In general, W1 quickly diffuses away from its original position (within ~2.3 ns; Supplementary Table 2) but other water molecules can take its place (*vide infra*). With regards to sfGFP, W1 is H-bonded to the chromophore for just under 6% of time (Supplementary Table 2). In comparison, W1 H-bonds to the chromophore more frequently in the H148S (19.6%), H148A (22.6%) and H148T (13.1%) mutant simulations. Both forms of H148N together with H148C model simulations show similar H-bond occurrence as sfGFP WT (Supplementary Table 2). Therefore, the short time scale MD modelling data suggests that the H148S mutation has the highest propensity to populate local H-bonds with the chromophore phenolate group.

### The effect of mutating H148 on the spectral properties of sfGFP

Based on our modelling, we generated H148A, H148C, H148N, H148S and H148T mutants of sfGFP to confirm how our simulation-based modelling approach can help predict the impact of mutations on polar interactions with the Cro. The H148T mutant in our hands did not generate any fluorescent protein suggesting the mutation impacted folding and/or chromophore maturation. H148A and H148C results in a mixed population comprising a dominant CroOH peak (~400 nm) and minor CroO^-^ peak (~490 nm) (Fig. 1c, Supplementary Fig. 4a and Supplementary Table 3). This confirms that removing the potential to form a H-bond between residue 148 and the Cro shifts the majority chromophore form sampled to CroOH. It was thought that residue 65 comprising the chromophore was the prime driver of chromophore ground state charge^40^. Both variants emit at ~510 nm irrespective of excitation at either ~400 nm or ~490 nm (Supplementary Fig. 4b–d and Supplementary Table 3), with excited state proton transfer being the likely mechanism responsible for the large Stokes shift between the CroOH excitation and the emission wavelength^22^. The H148N sfGFP mutant had a similar absorbance spectrum to WT sfGFP (Supplementary Fig. 4d), so retaining the CroO^-^ ground state. Previous work had suggested that H148N should promote the CroOH despite being able to make H-bond contact with the phenol hydroxyl group^41^. The overall spectral properties of sfGFP-H148N are no better than the parental sfGFP (Supplementary Table 3), so this mutant was not explored further. The short time scale dynamics may offer an explanation. The modelling showed that the orientation of the carboxamide essentially dictates H-bonding to the Cro phenolate (Supplementary Fig. 3 and Supplementary Table 1). The asparagine side chain can adopt a large number of rotamermic forms (12 forms for N148 based on PyMOL^42^ modelling) many of which will not form H-bonds with the phenolate oxygen. The likely conformational heterogeneity of N148 coupled with a lower propensity of sfGFP H148N to form a H-bond between the Cro and the structurally conserved water molecule (Supplementary Table 2) may be the basis behind the lack of overall functional improvement compared to sfGFP.

The H148S mutation results in a variant that has higher molar absorbance and QY compared to sfGFP, with the variant being 1.5 fold brighter than sfGFP itself (Fig. 1c, Tables 1) and 1.7 times brighter than EGFP. The sfGFP H148S protein, termed YuzuFP from here on, has a slightly red shifted excitation and emission (Table 1), giving the protein a yellow hue compared to sfGFP, similar to the Yuzu fruit. YuzuFP has a Stokes shift of 19 nm, which is larger than the recently developed StayGold^30,43,44^ variants (7–10 nm), meaning there is less spectral overlap between the peak excitation and emission events. The fluorescence lifetime of YuzuFP (2.76 ns; Supplementary Fig. 5a) is also slightly longer than both sfGFP (2.51 ns; Supplementary Fig. 5a) and EGFP (2.6 ns). Mutation of H148 does not significantly affect the chromophore’s pKa (6.2), which is similar to sfGFP and EGFP (Table 1 and Supplementary Fig. 5b). This suggests that H148 is not playing a major role in dictating pH dependent avGFP fluorescence. The maturation rate of YuzuFP is similar to sfGFP (Supplementary Fig. 5c); when standardising our results to the reported value for sfGFP (13.6 min)^14^, YuzuFP maturation time is 15.9 min. YuzuFP fluorescence was not adversely affected by salt (NaCl or KCl; Supplementary Fig. 5d–g) and is also largely monomeric in line with the parental sfGFP (Supplementary Fig. 6).

**Fig. 2:**
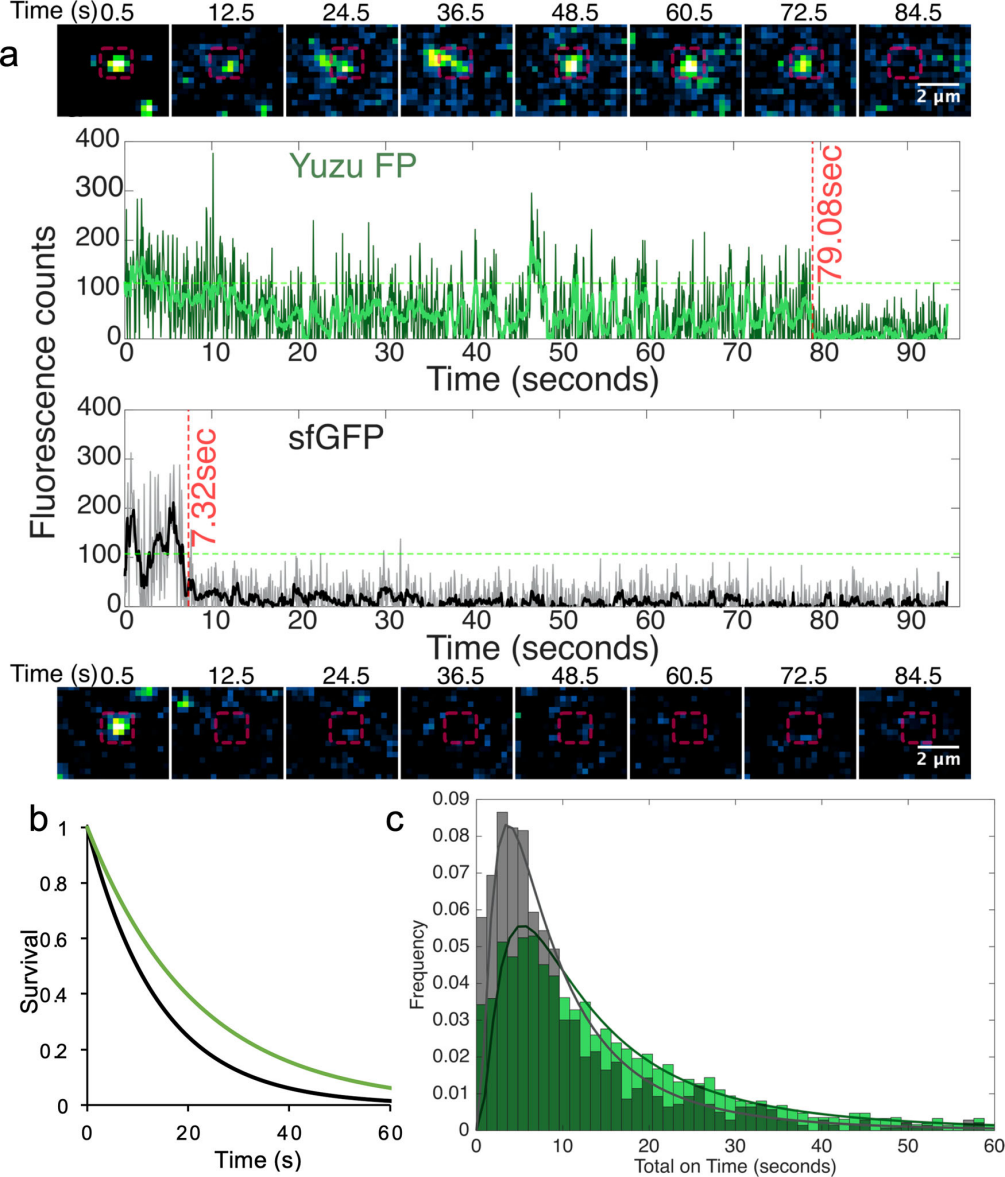
Single molecule fluorescence characteristics of sfGFP and YuzuFP. **a** Example single FP fluorescence images at various timepoints and their corresponding emission traces for YuzuFP (top, green) and sfGFP (bottom, black). In the image time course, the red box denotes the region of interest representing the analysed area. Dark green and grey traces show the raw data for each protein while the lighter green and black lines show the denoised data after applying a Chung-Kennedy filter. The green dashed line in each instance represents the threshold separating values considered on and off. More traces can be found in Supplementary Fig. 7. **b** Single molecule photobleaching survival plot for YuzuFP (green) and sfGFP (black) based on proportion of FPs retaining fluorescence (survival) over time. The decay curves were generated by fitting a single component exponential function to empirical cumulative distribution functions comprised of lifetimes from 3766 and 1283 individual FP traces for Yuzu FP and sfGFP, respectively. **c** Frequency distribution of total “on” times for YuzuFP (green) and sfGFP (grey), representing the cumulative time molecules across each population spent in an “on” bright state prior to photobleaching. The data was binned at 1.2 s intervals. The solid lines represent a log-normal fit to the distribution. Source data for plots in (**a**–**c**) are provided in Supplementary Data 1.

FP photostability as well as high fluorescence intensity is desirable for super-resolution techniques as it allows imaging under extreme illumination conditions such as stimulated emission depletion (STED) and total internal reflection fluorescence (TIRF) microscopy approaches, but also allow good fluorescent contrast where photobleaching limits the ability to resolve signal from background^45^. We next undertook single FP analysis of sfGFP and YuzuFP using TIRF microscopy^46^. As well as it’s increased brightness, YuzuFP has improved phototstability at the single molecule level compared to sfGFP; comparison of individual traces shows that overall YuzuFP remains fluorescent over longer time scales than sfGFP (Fig. 2a and Supplementary Fig. 7, Table 1). This equates to YuzuFP being more resistant to photobleaching (Fig. 2b) with an ensemble half-life under our single molecule imaging conditions of 15.0 ± 0.1 s, approximately two fold longer than WT sfGFP (half-life 8.3 ± 0.1 s). In line with the increased half-life, the lifetime increases from 12.0 ± 0.2 s for sfGFP to 21.5 ± 0.1 s. Analysis of individual traces show that a significant number of YuzuFP molecules (24%) remained fluorescent for >25 s (Fig. 2a and Supplementary Fig. 7) at sufficient laser powers for dynamic single molecule imaging at a time resolution of 60 ms (see Methods). As sfGFP is already one of the most photostable green-yellow FPs^15^, YuzuFP represents a marked improvement (see Table 1 for examples). The exact nature by which the H148S mutation improves resistance to photobleaching is currently unknown. One possible explanation is that the improved H-bonding may make the YuzuFP chromophore environment less suspectable to conformational changes that can occur on illumination^47^ or stall photochemical modification events^48^. The H148S mutation may also reduce the frequency of access of secondary molecules (e.g. oxygen) to the chromophore pocket that can cause photochemical modification (*vide infra*).

FPs observed at the single molecule level have a propensity to “blink”, i.e. switching between “on”/bright and “off”/dark states. Previously the presence and type of this blinking has been indicated as a contributing factor in the manifestation of ensemble level behaviours. YuzuFP is shown to cumulatively spend a longer time in its on state than sfGFP with the mean on-time peak shifting from 3.6 s to 5.3 s (Fig. 2c).

**Fig. 3:**
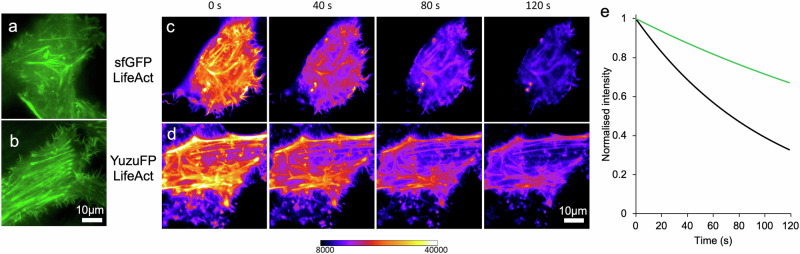
Live cell imaging of YuzuFP and sfGFP LifeAct Fusions. Wide-field image of **a** sfGFP and **b** YuzuFP LifeAct fusions. Time course of **c** sfGFP and **d** YuzuFP LifeAct fusions false coloured using a 16 bit Fire look-up table with the black value set to 8000, and the white value set to 40,000, to visualise signal within the dynamic range of the microscope setup. Note the similar intensities following 40 s constant exposure of sfGFP, to the 120 s timepoint of YuzuFP. **e** In situ photobleaching of YuzuFP (green line) and sfGFP (black line). Six independent intensity values were normalised to 1.0 (based on the intensity at 0 s), background subtracted and fit to a one phase decay curve in GraphPad Prism. Source data for plots in (**e**) are provided in Supplementary Data 1.

To confirm that in vitro performance translates to in situ cell imaging, a Lifeact-YuzuFP fusion was imaged by wide-field fluorescence microscopy in live cells. HeLa cells expressing the LifeAct fusions clearly show actin filament structures as expected (Fig. 3a, b). While the ability to resolve filament structure is lost at 40 s for sfGFP-LifeAct fusions, YuzuFP-LifeAct retains the ability to visualise clear filaments structures up to 120 s in line with the improved photostability. The photobleaching half-life is calculated to be nearly 3-fold higher for the YuzuFP fusion compared to sfGFP (206 ± 3.7 s versus 74 ± 0.5 s, respectively) with corresponding increases in lifetimes (106 ± 0.7 s for sfGFP and 297 ± 5.2 s for YuzuFP) confirming YuzuFP’s superior photostability over the already stable sfGFP starting point (Table 1).

### Long time scale molecular dynamics reveals potential beneficial effect of H148S mutation

**Fig. 4:**
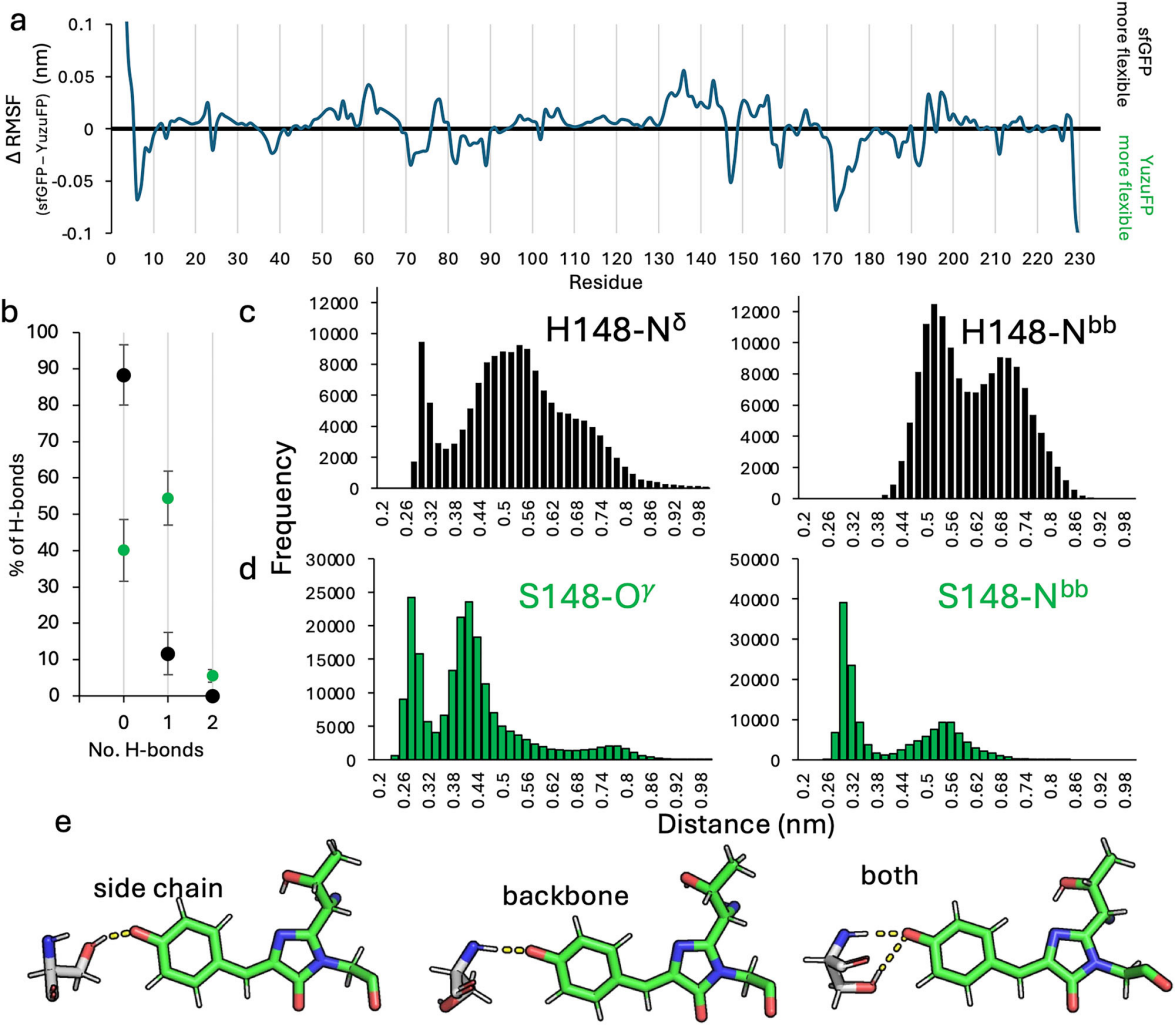
Hydrogen bonding between the chromophore and residue 148. **a** Per residue Cα root mean square fluctuation (RMSF) difference plot. The difference plot was generated by subtracting the Cα RMSF values of YuzuFP from that of sfGFP. The individual RMSF plots are shown in Supplementary Fig. 8a. Positive values indicate that sfGFP is more flexible and negative values that YuzuFP is more flexible. **b** The percentage of time H-bonds are formed between Cro and residue 148 over the course of the MD simulations for sfGFP (black) and YuzuFP (green). Error bars are the standard deviation between values measured for the 3 individual simulations. The pair-wise distance distribution in **c** sfGFP and **d** YuzuFP, between chromophore phenolate oxygen and residue 148 backbone and side chain H-bond donor heavy atoms across all simulation data. Bin sizes are 0.02 nm. The distance against time plot is shown in Supplementary Fig. 9. **e** Representative individual trajectories illustrating the H-bond between the S148 side chain hydroxyl group (simulation 2, 297.80 ns), backbone amine group (simulation 2, 254.39 ns) or both in YuzuFP (simulation 2, 5.43 ns). H-bonds are shown as orange dashes. Source data for plots in (**a**–**d**) are provided in Supplementary Data 1.

To investigate how the H148S mutation could be exerting its influence, we extended the initial MD simulations to three repeats of 500 ns on the YuzuFP CroO^-^ model and compared to sfGFP. Comparison of root mean square fluctuation (RMSF) shows both proteins display relatively similar backbone fluctuations (Supplementary Fig. 8a), with most differences being on the sub Ångstrom level (Fig. 4a). The region comprising the loop preceding residue 148 and strand following it (residues 145–151) shows some difference in flexibility between YuzuFP and sfGFP but the ΔRMSF is small, generally lower than 0.05 nm (Fig. 4a). The chromophore does not undergo any major structural change over the course of the simulation for both sfGFP and YuzuFP (Supplementary Fig. 8b, c). The RMSF of the hydroxyethyl group of the original T65 together with the original backbone oxygen of G67 show some fluctuation but most Cro atoms remain relatively unperturbed (Supplementary Fig. 8d).

One major difference is the interaction between Cro and residue 148. With regards to sfGFP, H-bonding occurs less frequently with H148 in sfGFP than S148 in YuzuFP. H148 of sfGFP does not make H-bond contact with the chromophore for *circa* 88% of the simulation time while in YuzuFP, the S148 H-bonds with Cro for *circa* 60% of the simulation time, with two H-bonds forming for ~5.5% of the time (Fig. 4b). This is similar to that observed over the initial 10 ns simulations (Supplementary Table 1). The change in H-bond frequency manifests in the pairwise distance distribution between the Cro phenolate O atom and either the side chain (Nδ atom in sfGFP H148 or Oγ atom in YuzuFP S148) or the backbone amide N (Fig. 4b, c). The side chain of YuzuFP’s S148 H-bond group is on average 0.1 nm closer to the Cro phenolate oxygen than H148 in sfGFP (0.42 nm ±0.13 versus 0.52 nm ±0.14). It is also clear from the atom pair distance distributions that the backbone amide N of S148 in YuzuFP is more consistently within H-bond distance of the Cro phenolate oxygen (<0.35 nm) while H148 in sfGFP rarely comes within 0.4 nm (Fig. 4c, d); the average atom pair distance between the amide nitrogen and the chromophore phenolate oxygen being 0.40 ± 0.12 nm in YuzuFP compared to 0.61 ± 0.10 nm for sfGFP. The backbone amide group of S148 in YuzuFP can form a H-bond with the chromophore phenolate oxygen, which is not observed for sfGFP. Indeed, on average just over 50% (55% ±21) of H-bonds between S148 and the chromophore phenolate oxygen involve the backbone amide. This suggests that introduction of the H148S mutation allows either the side chain or backbone to H-bond with the chromophore phenolate oxygen, and for 5% of the time, both. This is clearly seen from individual trajectories whereby either the side chain, the backbone amide or both are forming H-bonds with the Cro phenolate oxygen (Fig. 4e).

**Fig. 5:**
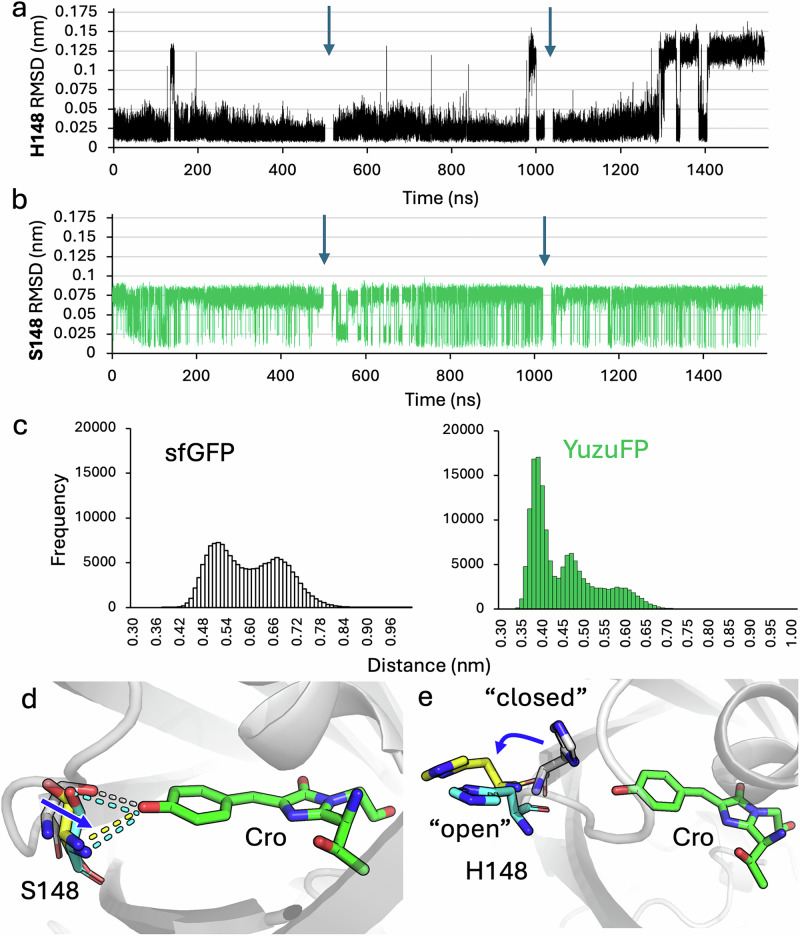
Conformational changes to residue 148. The root mean square deviation (RMSD) of **a** H148 in sfGFP and **b** S148 in YuzuFP. **c** The distance distribution between the Cro phenolate oxygen and the Cα of residue 148 for all simulations. The bin size is 0.01 nm. The distance change over time plot for each simulation is shown in Supplementary Fig. 10. **d** Representative trajectories of YuzuFP (grey, 0 ns; yellow, simulation 1, 150.75 ns, RMSD 0.09 nm; cyan, simulation 1, 168.74 ns, RMSD 0.08 nm). The dashed lines represent polar contacts as determined using PyMOL^42^. **e** Representative trajectories of sfGFP (grey, 0 ns; yellow simulation 3, 262.86 ns, RMSD 0.12 nm; cyan, simulation 3, 250.87 ns RMSD 0.12 nm). The blue arrow indicates the direction of backbone movement with respect to the starting trajectory (0 ns). Source data for plots in (**a**–**c**) are provided in Supplementary Data 1.

The reason for the change in H-bond interactions appears to be largely down to changes in orientation and distance of residue 148 with respect to the chromophore. The root mean square deviation (RMSD) for H148 in sfGFP for most of the simulations appears to be relatively stable but periodically undergoes a significant change (>0.1 nm; Fig. 5a). The RMSD of S148 in YuzuFP quickly converts to a form with an RMSD of *circa* 0.08 nm and fluctuates periodically with a conformation closer to that of the starting conformation (Fig. 5b). Distances between the chromophore phenolate oxygen and the Cα of residue 148 suggest that in YuzuFP, the change in RMSD can be ascribed to S148 moving closer to the chromophore while H148 in sfGFP moves further away (Fig. 5c); the average distance between the 148 Cα and chromophore phenolate oxygen is 0.45 nm ±0.08 nm for YuzuFP, circa 0.15 nm shorter than in sfGFP (0.59 nm ± 0.08). The higher RMSD observed for YuzuFP does not impact S148’s ability to form H-bonds with the chromophore (Fig. 5d). The larger periodic deviations observed for H148 in sfGFP mirrors the alternative “open” conformation observed in crystal structures (Fig. 5e)^24,31,36,37^. Thus, the closer association of S148 with the chromophore together with a high propensity to form H-bonds is likely to play a key role in improving the properties of YuzuFP.

There may also be additional structural changes that could also contribute to YuzuFP’s improved properties. For example, F145, which is located nearby to residue 148, was mutated from tyrosine on generating sfGFP and is known to improve stability and shielding of the chromophore from solvent^14,49^. In YuzuFP, F145 is more closely associated with the chromophore than in sfGFP with the average F145 aromatic Cζ to Cro Cζ (Cz in Supplementary Fig. 8d) distance being 0.57 nm (±0.07) compared to 0.90 nm (±0.26) for sfGFP. Thus, the H148S mutation may change the local structure and dynamics beyond the direct contact with the chromophore, possibly resulting in beneficial effects.

### Dynamic water molecules

**Fig. 6:**
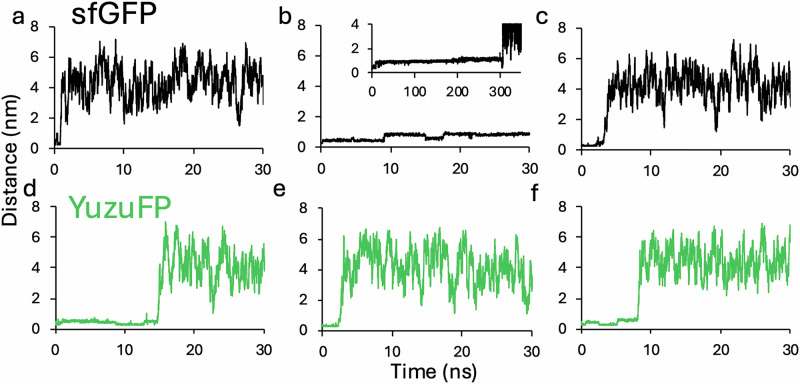
Residency of the structurally the conserved water molecule, W1, close to the chromophore phenolate oxygen. Distance between the chromophore phenolate oxygen and the O atom of the water molecule W1 (see Fig. 1a for reference) in (**a**–**c**) sfGFP and (**d**, **e**) YuzuFP in each of the 500 ns simulations. In (**b**), shown inset is the distance over an extended period (350 ns). Source data for plots in (**a**–**f**) are provided in Supplementary Data 1.

The chromophore, while being internal to the barrel structure, is well solvated with sfGFP having six water molecules surrounding the chromophore in the original crystal structure of sfGFP^14^ (Supplementary Fig. 11). One water molecule, termed W1 in Fig. 1b, is ever present in crystal structures of avGFPs and other FPs (Supplementary Fig. 1) forming polar contacts with Cro phenolate O as well as the side chain of S205; it’s positioning is thought to be important in promoting the Cro-O^-^^32,34^ state through proton shuttling and enhancing fluorescence^22,50–52^. Our MD simulations show that W1 is associated with the protein for a relatively short time at that spatial position in both sfGFP and YuzuFP before exchanging with bulk solvent (Fig. 6). The residency time of W1 for YuzuFP is longer (*circa* 8.5 ns) compared to sfGFP (*circa* 1.5 ns). The analysis is complicated as in one sfGFP simulation, W1 quickly becomes internalised (within 0.22 ns) close to the Cro (Fig. 6b and Supplementary Fig. 12). W1 appears to be replaced through the simulation with other water molecules; the Cro phenolate oxygen in YuzuFP spends only 5.7% of its time with no H-bonds to water, which increases slightly to 6.8% in sfGFP.

The remaining water molecules that reside within the barrel structure overall have longer residency times but most eventually undergo bulk exchange with the solvent (Supplementary Fig. 13). Analysis of potential tunnels using CAVER^53,54^ reveals an exit point close to H148 in sfGFP (Supplementary Fig. 14) providing a potential route to exchange with bulk water. However, some of these water molecules reside close to the Cro over the course of simulation. For YuzuFP, the water molecule W2 remains buried in two simulations whereas it exchanges within 172 ns in sfGFP (Supplementary Fig. 13 and Supplementary Table 4). In the third YuzuFP simulation, two other water molecules (W5 and W6) remain buried over the 500 ns (Supplementary Fig. 13 and Supplementary Table 4). In contrast, only two waters in a single simulation remain buried over the simulation for sfGFP (Supplementary Fig. 13 and Supplementary Table 4). Water exchange rates with the bulk solvent could be indicative of accessibility to the chromophore pocket by other solutes such as reactive oxygen species that can subsequently cause photobleaching through covalent modifications and disruption of bond networks^48^. Thus, mutations to residue 148 that affect local dynamics could also influence tunnel aperture and thus access to the chromophore pocket.

## Conclusion

The avGFP still provides the basic framework by which many FPs are engineered, but H148 remains largely a constant in green-yellow versions despite FPs from other origins using different residue types in the same capacity. Using a molecular dynamics-based approach we were able to model the potential changes to chromophore interactions on mutating H148 to all other natural amino acids in solution, something not achievable by classical protein design approaches. Short scale molecular dynamics suggested H148S mutation will generate a more persistent H-bond with the chromophore than the native histidine. By mutating H148 to serine in the sfGFP background, the engineered FP termed YuzuFP retains its fast-maturing properties and is brighter, more resistant to photobleaching, and stays on for longer making it a good candidate for modern fluorescence microscopy approaches. The origin of the improvement appears to be changes in the local dynamics around residue 148 with mutation to serine resulting in a more stable and persistent interaction with the chromophore and water molecules. While we expect YuzuFP to retain the highly stable nature of sfGFP with its fast-folding kinetics, future work will involve investigating the impact of H148S on these critical properties. Thus, YuzuFP could prove to be an important tool for researchers and together with MD-based design could form the basis for future protein engineering endeavours aimed at further improving its properties such as brightness. Indeed, given that we show here that H-bond persistency with the chromophore and chromophore solvation play a key role in improved probe performance, our approach could be applied to other regions of avGFP, other variants of avGFP and even FPs of a different origin to generate better FPs. Furthermore, as local interaction network dynamics and solvation play a vital role in determining the protein structure-function relationship in general, MD-based approaches could be used not only to understand fundamental molecular events but as a core part of the protein design process.

## Methods

### Protein engineering and recombinant production

The sfGFP variants in the pBAD plasmid were generated using a whole plasmid, inverse PCR process as described previously^31,32^ using the primers in Supplementary Table 5 and the Q5^®^ site-directed mutagenesis kit from New England Biolabs. Recombinant production was performed in *E. coli* essentially as described previously^31^. Briefly, chemically competent *E. coli* TOP10™ cells were transformed with the prerequisite plasmid and plated on LB agar plates supplemented with 50 μg/mL ampicillin. A single colony was taken from LB agar plate and used to inoculate a 10 mL 2xYT starter culture supplemented with and incubated overnight with shaking. The overnight culture was used to inoculate a 1 L culture of autoinduction media (see supporting methods). The culture was inoculated overnight at 37 °C with shaking. Cells were then pelleted by centrifugation and suspended in 50 mM Tris, pH 8.0. Cells were lysed using the French Pressure Cell. Soluble cell lysate was then separated from insoluble fractions via centrifugation using a Beckman JLA 25.50 rotor at 25,000 × *g* for 40 min. Protein purification was carried out with an ÄKTA Purifier FPLC using columns purchased from Cytiva and protein elution monitored at 280 nm, 400 nm and/or 485 nm. Clarified cell lysate was passed through a 5 mL His Trap^TM^ HP column (binding capacity ~200 mg protein) equilibrated in 50 mM Tris, pH 8.0 buffer containing 10 mM imidazole. Bound target protein was then eluted by the addition of the elution buffer containing imidazole at a gradient from 10 to 500 mM imidazole. Fractions were checked for purity via SDS-PAGE analysis. Finally, the proteins were further purified by size exclusion chromatography (SEC) using a HiLoad^TM^ 16/600 Superdex^TM^ S75 pg column (Cytiva) equilibrated with 50 mM Tris, pH 8.0. Protein samples were concentrated using Vivaspin^TM^ 10 kDa molecular weight cut-off spin filters (VWR) by centrifugation at 4000 × *g* until the desired volume is reached. Analytical SEC was performed using a Superdex 75 10/300GL column (Cytiva). For maturation analysis, an approach similar to that described by Shaner et al. was used^17^. An overnight LB liquid culture was diluted to a final OD_600_ of 0.5 in a 5 mL culture tube filled to the brim and the cap sealed with parafilm to minimise aeration. The cultures were incubated for 5 h at 37 °C while shaking. Cells were pelleted by spinning in a microfuge at 13,000 rpm for 5 min at 4 °C. The weight of cell pellets was determined and 500 μL of B-PER™ Complete Bacterial Protein Extraction Reagent (ThermoFisher Scientific) without any nuclease or lysozyme was added per 100 mg of cell pellet and left on ice for 10 min with occasion tube inversion. The lysate was centrifuged for 10 min at 13,000 rpm in a microfuge at 4 °C. Three individual samples were prepared ready for spectral analysis. It should be noted that YuzuFP producing cultures produced a noticeable fluorescent cell pellet even under limiting O_2_ conditions while those producing sfGFP remained largely colourless.

### Spectroscopic analysis

Protein concentrations were determined using the Bio-Rad DC Protein Assay using sfGFP as the standard. Concentration was confirmed by comparison to sfGFP’s 280 nm absorbance (ε_280_ = 25.3 mM^−1^cm^−1^); as the mutants did not contain any additional UV absorbing side chains their 280 nm molar absorbance should be the same as for sfGFP (see Fig. 1c). UV–visible (UV–vis) absorption spectra were recorded on an Agilent Cary 600 spectrophotometer in a 1 cm pathlength cuvette. Spectra were recorded from 200 to 600 nm at a rate of 300 nm/min using a known protein concentration (usually 5 μM) and the Beer–Lambert equation used to determine the molar absorbance of each variant protein. Emission and excitation spectra were measured using a Varian Cary Eclipse Fluorimeter with a QS quartz cuvette (Hellma). Data were collected with 5 nm slit width at a rate of 300 nm/min. Emission spectra were recorded at a fixed excitation wavelength according to the excitation maximum of the variant. Spectra were recorded using 0.5 μM of protein in 50 mM Tris-HCl, pH 8.0. Quantum yield was determined as described previously^23^ using a fluorescein standard (QY = 0.75 in 0.1 M NaOH). Maturation rates were measured by immediately diluting the B-PER™ lysed solution after the centrifugation step 4–6 fold in 50 mM Tris HCl, pH 8.0. Emission at 512 nm on excitation at 485 nm was measured every minute over 90 min. Each sample was left in the dark for >10 h and the final end point measurement was taken to determine the final fluorescence value (F_max_) together with an emission spectrum. Measurements were done in triplicate. The pKa was determined using changes in absorbance at the λ_max_ equivalent to the CroOH (~400 nm) and CroO^-^ (~490 nm) using the following buffers: 100 mM glycine-HCl (pH 3.0), 100 mM acetate buffer (pH 4.5–5.5), 100 mM KH_2_PO_4_-NaOH (pH 6.0), 100 mM HEPES buffer (pH 7.0 and 8.0), 50 mM TrisHCl (pH 8.0), 100 mM glycine-NaOH (pH 9.0–10.0), 100 mM Na_2_HPO_4_-NaOH (pH 11–12). Fluorescence lifetime (Tau) measurements were performed using a bespoke TetherScan device setup^55^ at protein concentrations ranging from 5 to 15 μM. The resulting decay curves were fit to a single exponential decay using GraphPad Prism equation (*F* = *F*_*0*_ + (*F*_*min*_ − *F*_*0*_)⋅ e^−k⋅t^) where t is time in ns, *F* is fluorescence signal, *F*_*0*_ is fluorescence signal at time 0, *F*_*min*_ is the minimum value reached as t reaches infinity, and k is the rate constant).

### Single molecule analysis

Single molecule imaging of YuzuFP and sfGFP was carried out similar to that as described previously^32^. Briefly, total internal reflection fluorescence (TIRF) imaging of a low concentration, non-diffusing FP solution in an aqueous thin film, on a plasma treated coverslip was performed using a custom optical setup. The core of this is a Nikon Ti-U inverted microscope with a high numerical aperture 60× objective (Nikon, CFI Apochromat TIRF) and an Andor iXon ultra 897 camera. Excitation was achieved using a fibre-coupled Venus 473 nm DPSS laser with a power output of 100 mW, achieving ~212.5 mW/cm^2^ at the sample focal plane (as measured using a photodiode and powermeter (S121C and PM130D, Thorlabs). A 488 nm dichroic mirror coupled with a 500 nm long pass filter and a 525/50 nm bandpass filter were used to isolate the fluorescence signal. Acquisitions were made in areas without prior laser exposure to minimise the effects of photobleaching prior to image capture. An exposure time of 0.06 s and an acquisition period of 96 s (1600 frames) was used in each experiment. Acquired image stacks were processed using the FIJI distribution of ImageJ to normalise for laser power fluctuation and spatial variations in laser intensity as previously described^32,56^. Detection and extraction of single molecule time series data was also carried out in FIJI using the TrackMate plugin^57,58^ employing a difference of gaussians detection method and an estimated spot size of 4 pixels. Extracted time series were exported to MATLAB and a forwards backwards moving window Chung Kennedy filter^59^ was applied to aid in data set observation. An algorithmic approach was then developed to identify ”on” (bright) and ”off” (dark) states in individual data sets by first identifying the photobleaching time and then looking for instances prior to this event where values surpassed a given intensity threshold for a period longer than a given temporal window. In brief, an intensity threshold for each time series was determined by first segmenting using a minimum sum log-likelihood deviation from segmented means method^60^. Values from the lowest intensity segment, taken to be the photobleached, background state, determined by this method were averaged. An intensity threshold was determined by exploring different multiples of standard deviation from the mean to identify a case where mis-detection of events in a given lowest intensity segment was >0.1% and where mis-detections across all data sets occured in >2% of the population. From this process the identified threshold was ten standard deviations from the mean. A second round of temporal thresholding was applied to values meeting the intensity threshold criteria. A window size of 15 frames (0.9 s) was used as a lower limit to separate on-state events from background fluctuations in noise. Intensity values showing sequential temporal separation below this threshold were grouped into single ”on” blinking events. Photobleaching lifetime was determined as the end point of the last of these events in a given time series. Cumulative on-time was taken as the sum of all on-events within a given molecule’s photobleaching lifetime. Mean on-time of the two species was determined by fitting a log-normal distribution to a probability density function generated from all on-times from each FP using GraphPad Prism. Similarly, the survival half-life was determined by fitting a single component exponential decay to the empirical cumulative distribution function of photobleaching times for each FP. Datasets for the two FPs were made up of three experimental repeats each and a total of 3766 and 1283 single molecule spots and corresponding time series were extracted from the YuzuFP and sfGFP datasets respectively.

### Cell imaging

The LifeAct-sfGFP fusion gene was synthesised by Twist Biosciences (see Supporting Information for sequence) and placed in their pTWIST CMV mammalian expression vector. Human cervical carcinoma (HeLa) cells (ATCC, UK) were cultured directly onto live cell dishes (Mattek, USA), and allowed to adhere overnight before transfecting using Fugene6 (Promega) according to manufacturer’s instruction using 1 μg of DNA per 3 μL Fugene in a volume of 100 μl of OptiMEM (Life Technologies, UK). Before imaging, cells were washed and the media replaced with Fluorobrite DMEM containing 10% FBS (Life Technologies, UK). Wide-field epi fluorescence measurements were conducted on an inverted Olympus IX73 microscope and a Prior Lumen200Pro light source using filter set (89000, Chroma, Vermont, U.S.A.) selecting the ET490 nm/20 nm as the excitation filter and the ET525 nm/36 nm as the emission filter. The fluorescence emission was detected with a Hamamatsu ORCA-flash 4.0 V2 sCMOS Camera operated utilizing the HCImage software package (Hamamatsu). A 100× oil immersion objective with an NA of 1.4 was used to collect sequential images of 1 s exposure over 121 timepoints. Cells were imaged live at 37 °C 5% CO_2_ in a humidified chamber.

For quantification of intensity and photobleaching, a 10 μm diameter circular region of interest was measured for average intensity within individual transfected cells utilizing the multi measure function within the ROI manager of FIJI^56^. All regions of interest were checked to ensure that all pixels were within the dynamic range of the microscope setup. All images were captured and processed using the same methodology. >20 ROIs per condition were assessed. Fluorescence intensity measurements were normalized to their brightest value (at timepoint 0), background adjusted to fit to a single phase decay model in GraphPad Prism.

### Modelling and molecular dynamics

For the short scale MD, the initial models of every possible amino acid at position 148 were generated using the PyMOL^42^ mutagenesis tool using the sfGFP crystal structure (PDB 2b3p) as the starting point. Short time scale molecular dynamics were performed using GROMACS^61,62^ with a AMBER99SB forcefield^63^ modified to contain constraints for the GFP chromophore in the Cro-O^-^ form (see https://github.com/drdafyddj/GFP). The protein was then placed centrally in a cubic box at least 1 nm from the box edge (8.58346 nm × 8.58346 nm × 8.53468 nm) applying periodic boundary conditions. The protein system was then solvated with water molecules (TIP3P) and total charge balanced to zero with Na^+^ ions. The protein was then energy minimized to below 1000 kJ mol^−1^ nm^−1^ with an energy step size of 0.01 over a maximum of 50,000 steps. The system was then temperature and pressure equilibrated using the NVT (constant number of particles, volume and temperature) followed by NPT (number of particles, pressure and temperature) ensembles. MD runs were then performed at 300 K, 1 atmosphere pressure for 10 ns with a 2 fs time step integration. Clustered average models sampling the duration of the simulations were used as representative structures of the mutations. Long scale molecular dynamics of sfGFP and YuzuFP were undertaken in the same manner but extended to 3 × 500 ns simulations. The original crystallographic waters present in the 2b3p PDB file were included in the long run simulations. All simulations were performed on the Super Computing Wales Hawk facility (project code scw1631). The protein in the trajectories was centred in the simulation box and dumps of individual trajectories were performed via the *trjconv* command. RMSD and RMSF calculations were performed using the *rms* and *rmsf* commands. Pairwise distances and hydrogen bonds were determined using the *pairdist* and *hbond* commands. The recommended *hbond* default parameters were used (https://manual.gromacs.org/current/onlinehelp/gmx-hbond.html).

### Reporting summary

Further information on research design is available in the Nature Portfolio Reporting Summary linked to this article.

## Supplementary information

Supplementary Information

Description of Additional Supplementary Files

Supplementary Data 1

Supplemenrary Data 2

Reporting Summary

## Data Availability

Source data are provided with this paper as Supplementary Data 1 (main manuscript) and Supplementary Data 2 (SI data). The molecular dynamic trajectory files for sfGFP and YuzuFP are available on request. The initial models are available via FigShare (https://figshare.com/s/ace6f0e9c9bba4618b1c). The YuzuFP sequence has been allocated the GenBank accession number PV666611. The original pBAD YuzuFP construct will be available via Addgene (ID 240149). Protein structures used in this work include PDB ID 2b3p (10.2210/pdb2B3P/pdb).

## Code availability

Molecular dynamics was performed using the open source software Gromacs (https://www.gromacs.org). Additional parameter files related to the sfGFP chromophore are available via FigShare (https://figshare.com/s/ace6f0e9c9bba4618b1c).
