# Supplementary material for: Molecular dynamics guided identification of a brighter variant of superfolder Green Fluorescent Protein with increased photobleaching resistance
Source: Commun Chem. 2025 Jun 5;8:174. doi: 10.1038/s42004-025-01573-4 (PMC12141695; doi:10.1038/s42004-025-01573-4)
Supplement: Supplementary file 1 — Supplementary Information [file 42004_2025_1573_MOESM1_ESM.pdf]

Molecular dynamics guided identification of a brighter variant of superfolder Green Fluorescent Protein with increased photobleaching resistance

Rochelle D. Ahmed[1,3], W. David Jamieson[2,3], Danoo Vitsupakon[1,3], Athena Zitti[1,3], (joint first authors), Kai A. Pawson[1], Oliver K. Castell[2], Peter D. Watson[1], D. Dafydd Jones[1]*.

Supplementary Information.

Supporting Methods.

Autoinduction media

The following lists the auto-induction media used to produce sfGFP proteins present in the pBAD plasmid: 1% (w/v) tryptone; 0.5 %(w/v) yeast extract; 0.5% (v/v) glycerol; 0.05 % (w/v) glucose; 0.2% lactose; 25 mM Na_2HPO_4; 25 mM KH_2PO_4; 50 mM NH_4Cl; 5 mM $NaSO_4$; 2 mM $MgSO_4$; 1 x trace metals (4 µM $CaCl_2$; 2 µM, $MnCl_2$, 2 µM $ZnSO_4$, 0.4 µM $CoCl_2$, 0.4 µM $CuCl_2$, 0.4 $NiCl_2$, 0.4 uM Na_2MoO_4, 0.4 µM H_3BO_3 and 10 µM $FeCl_3$ in ultra-pure water) and 0.05 % (w/v) L-arabinose. The medium was supplemented with 50 µg/mL ampicillin. All chemicals were supplied by Melford.

sfGFP gene sequence.

ATGGTTAGCAAAGGTGAAGAACTGTTTACCGGCGTTGTGCCGATTCTGGTGGAACTGGATGGTGAT
GTGAATGGCCATAAATTTAGCGTTCGTGGCGAAGGCGAAGGTGATGCGACCAACGGTAAACTGACC
CTGAAATTTATTTGCACCACCGGTAAACTGCCGGTTCCGTGGCCGACCCTGGTGACCACCCTGACCT
ATGGCGTTCAGTGCTTTAGCCGCTATCCGGATCATATGAAACGCCATGATTTCTTTAAAAGCGCGAT
GCCGGAAGGCTATGTGCAGGAACGTACCATTAGCTTCAAAGATGATGGCACCTATAAAACCCGTGC
GGAAGTTAAATTTGAAGGCGATACCCTGGTGAACCGCATTGAACTGAAAGGTATTGATTTTAAAGA
AGATGGCAACATTCTGGGTCATAAACTGGAATATAATTTCAACAGCCATAATGTGTATATTACCGCC
GATAAACAGAAAAATGGCATCAAAGCGAACTTTAAAATCCGTCACAACGTGGAAGATGGTAGCGTG
CAGCTGGCGGATCATTATCAGCAGAATACCCCGATTGGTGATGGCCCGGTGCTGCTGCCGGATAAT
CATTATCTGAGCACCCAGAGCGTTCTGAGCAAAGATCCGAATGAAAAACGTGATCATATGGTGCTGC
TGGAATTTGTTACCGCCGCGGGCATTACCCACGGTATGGATGAACTGTATAAAGGCAGCCACCATCA
TCATCACCATTAA

LifeAct-sfGFP gene sequence

TCCGCCCCATTGACGCAAATGGGCGGTAGGCGTGTACGGTGGGAGGTCTATATAAGCAGAGCTGGT
TTAGTGAACCGTCAGATCCGCTAGCGCCACCATGGGCGTGGCCGACTTGATCAAGAAGTTCGAGTC
CATCTCCAAGGAGGAGGGGGATCCACCGGTCGCCACCATGGTGAGCAAGGGCGAGGAGCTGTTCA
CCGGGGTGGTGCCCATCCTGGTCGAGCTGGACGGCGACGTAAACGGCCACAAGTTCAGCGTGCGC
GGCGAGGGCGAGGGCGATGCCACCAACGGCAAGCTGACCCTGAAGTTCATCTGCACCACCGGCAA
GCTGCCCGTGCCCTGGCCCACCCTCGTGACCACCCTGACCTACGGCGTGCAGTGCTTCAGCCGCTAC
CCCGACCACATGAAGCGCCACGACTTCTTCAAGTCCGCCATGCCCGAAGGCTACGTCCAGGAGCGCA
CCATCAGCTTCAAGGACGACGGCACCTACAAGACCCGCGCCGAGGTGAAGTTCGAGGGCGACACCC

TGGTGAACCGCATCGAGCTGAAGGGCATCGACTTCAAGGAGGACGGCAACATCCTGGGGCACAAG
CTGGAGTACAACTTCAACAGCCACAACGTCTATATCACCGCCGACAAGCAGAAGAACGGCATCAAG
GCCAACTTCAAGATCCGCCACAACGTGGAGGACGGCAGCGTGCAGCTCGCCGACCACTACCAGCAG
AACACCCCCATCGGCGACGGCCCCGTGCTGCTGCCCGACAACCACTACCTGAGCACCCAGTCCGTGC
TGAGCAAAGACCCCAACGAGAAGCGCGATCACATGGTCCTGCTGGAGTTCGTGACCGCCGCCGGGA
TCACTCACGGCATGGACGAGCTGTACAAGTAAGCGGCCGCGACTCTAGATCATAATCAGCCATACCA
CATTTGTAGAGGTTTTACTTGCTTTAAAAAACCTCCCACACCTCCCCCTGAACCTGAAACATAAAATG
AA

Supporting Tables

Supplementary Table 1. H-bond frequency between chromophore and residue 148 over 10ns (1001 step) simulations

Variant	H-bonds = 0	H-bonds = 1	H-bonds = 2	% time H-bonding
sfGFP WT (H148)	952	49	0	4.9
sfGFP H148S	512	409	80	48.9
sfGFP H148T	993	8	0	0.8
sfGFP H148N [a]	222	779	0	77.9
sfGFP H148N [b]	992	9	0	0.9
sfGFP H148C	977	24	0	2.4
sfGFP H148A	1001	0	0	0

a, sfGFP H148N where starting rotamermic form has the NH group of the carboxamide group is closest to the chromophore phenol O.
b, sfGFP H148N where starting rotamermic form has the O group of the carboxamide group is closest to the chromophore phenol O.

Supplementary Table 2. H-bond frequency between chromophore and W1 water over 10ns simulations

Variant	H-bonds = 0	H-bonds = 1	H-bonds = 2	% time H-bonding	Time before distance > 0.4 nm[c]
sfGFP WT (H148)	945	56	0	5.6	0.22 ns
sfGFP H148S	805	196	0	19.6	2.24 ns
sfGFP H148T	870	131	0	13.1	1.4 ns
sfGFP H148N [a]	946	55	0	5.5	0.83 ns
sfGFP H148N [b]	931	70	0	7	0.1 ns
sfGFP H148C	973	28	0	2.8	0.53 ns
sfGFP H148A	775	224	2	22.6	1.05 ns

a, sfGFP H148N where starting rotamermic form has the NH group of the carboxamide group is closest to the chromophore phenol O.
b, sfGFP H148N where starting rotamermic form has the O group of the carboxamide group is closest to the chromophore phenol O.
c, refers to distance between the chromophore phenol O atom and the O atom of W1, with the quoted time based on the timepoint that the distance between the two atoms goes beyond 0.4 nm for a duration of at least 0.2 ns.

Supplementary Table 3. Spectral properties of sfGFP-H148X variants.

FP	λ_{max} (nm)	ε ($mM^{-1}cm^{-1}$)	λ_{EM} (nm)	QY	Brightness ($mM^{-1}cm^{-1}$)
sfGFP[a]	485	49.0	509	0.72	35.3
sfGFP^{H148C}	398	34.4	513	0.32	11.0
	498	11.1		0.50	5.6
sfGFP^{H148N}	487	54.9	509	0.37	20.3

a, originally reported by Reddington et al. [1]

Supplementary Table 4. Residency time of water molecules close to the chromophore.

	Residency time (ns)[a]							
	sfGFP				YuzuFP			
Water	Sim 1	Sim 2	Sim 3	**Average**	Sim 1	Sim 2	Sim 3	**Average**
W1	3.36	306.55 (0.22)[c]	0.89	**103.6 (1.49)**	14.79	8.15	2.64	**8.53**
W2	171.46	124.14	119.19	**138.26**	500[b]	63.83	500[b]	**354.61**
W3	33.54	200.19	500[b]	**244.58**	89.69	248.41	11.62	**116.57**
W4	50.77	188	119.19	**119.32**	37.14	162.39	2.88	**67.47**
W5	41.51	221.98	68.77	**110.75**	25.56	500[b]	42.15	**189.24**
W6	66.3	89.21	500[b]	**218.50**	73.43	500[b]	161.45	**244.96**

a, water residency time close to the chromophore as determined by a distance that is consistently (≥0.1 ns) above a distance of 1.5 nm from the β-methylene bridge atom.
b, retained residency close to the chromophore (see definition above) over the course of the 500 ns simulation. The average is thus calculated with these values set to 500 ns.
c, W1 in sfGFP Sim 2 moves from its original position to an internal position close to the chromophore. W1 becomes internalised within 0.22 ns (see Supplementary Fig. 11).

Mutation	Primer sequence (5' to 3')
H148S	CTGGAATATAATTTCAACAGC**AGT**AATG<br>TTTATGACCCAGAATGTTGCCATC
H148C	CGATAAACAGAAAAATGGCATCAAAGCG<br>GCGGTAATATACACATT**GCA**GCTGTTG
H148A	GGAATATAATTTCAACAGC**GCT**AATG<br>AGTTTATGACCCAGAATGTTGCCATCT
H148N	TTTCAACAGC**AAC**AATGTGTATATTAC<br>TTATATTCCAGTTTATGACCC
H148S (Mammalian constructs)	CAACTTCAACAGC**AGC**AACGTCTATATCAC<br>TACTCCAGCTTGTGCCCCAGGATGT

Supplementary Table 5. Primer combinations and sequences for introducing H148X mutations. Bases in bold and underlined represent the mutation. Top primer is the "forward" and bottom the "reverse" primer in a whole plasmid PCR.

Supporting Figures.

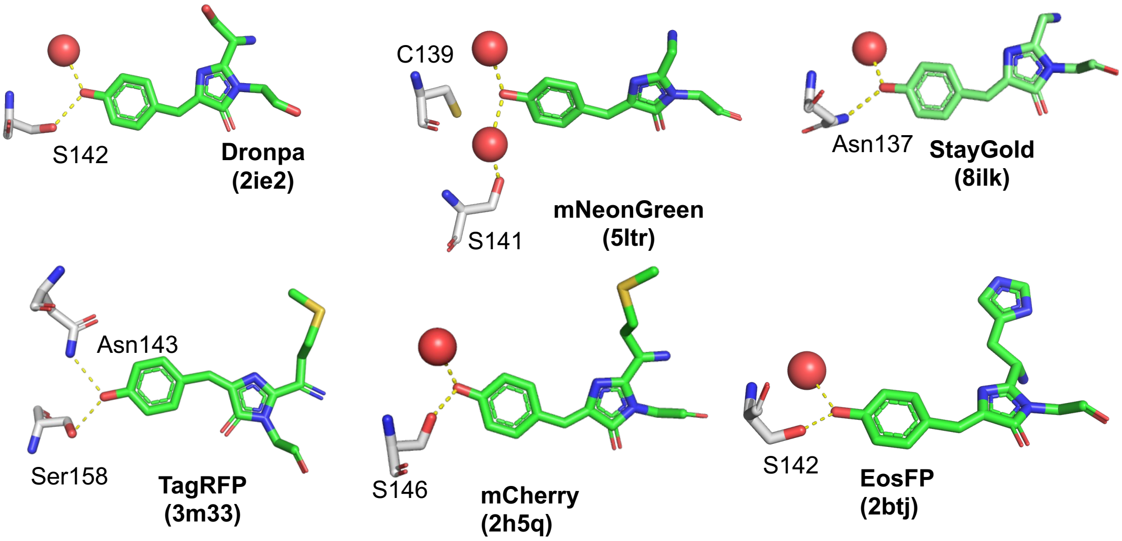

Supplementary Fig. 1. Additional examples of residues equivalent to H148 that interact with the chromophore phenol group: S142 in Dronpa [2] (PDB 2ie2), C139 and a secondary water molecule in mNeonGreen [3](PDB 5ltr), N137 in StayGold[4] (PDB 8ilk), N143 and S158 in TagRFP [5,6] (PDB 3m33), S146 in mCherry [7](PDB 2h5q), S142 in EosFP [8] (PDB 1zux). In all cases the chomophore is coloured green, interacting residues equivalent to H148 in sfGFP grey and water molecules are red spheres.

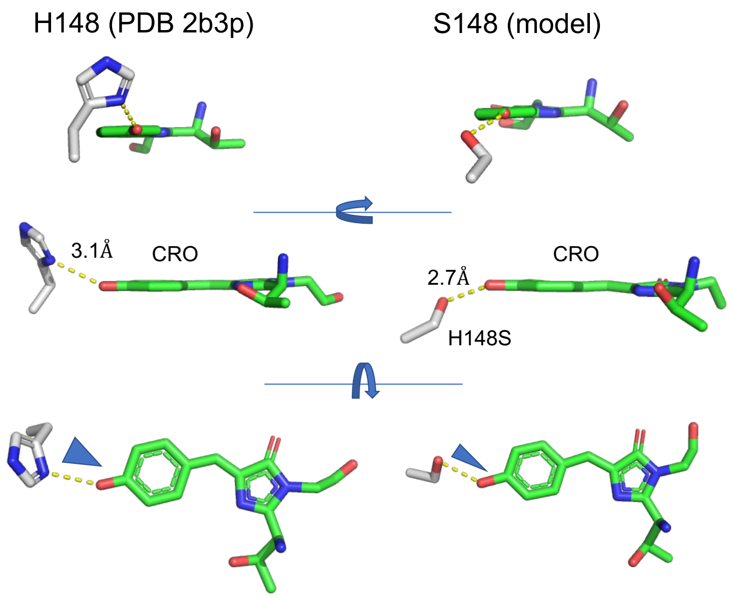

Supplementary Fig. 2. Relative conformations of residue 148 (grey sticks) and chromophore (green sticks). Left hand side is the position of H148 taken from the known structure of sfGFP (2b3p.pdb [9]). Modelled structure of the H148S is the clustered average of individual MD trajectory outputs after 10 ns of molecular dynamics.

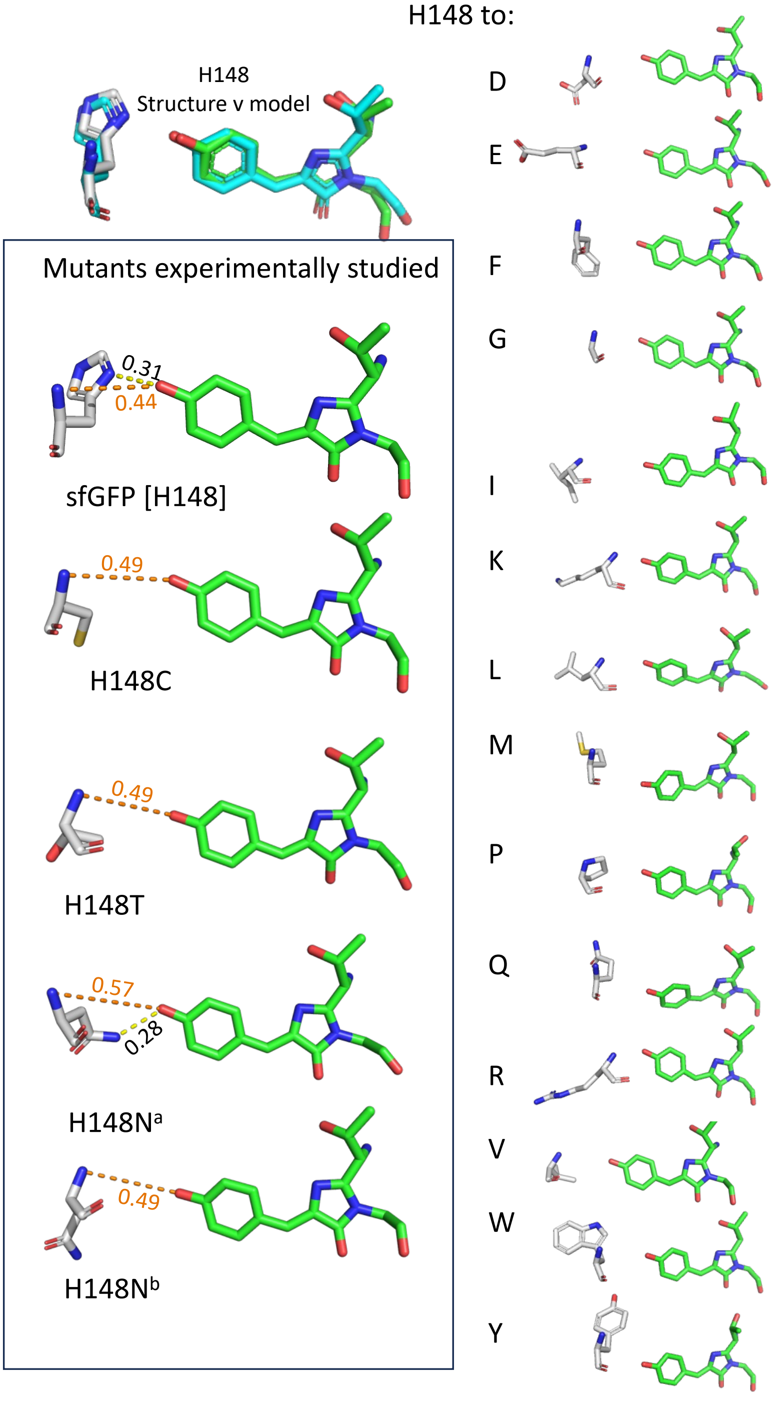

Supplementary Fig. 3. Modelling the H148X mutations. Models for sfGFP H148S and H148A are shown in the main manuscript (Figure 1b). Modelled structures of the H148X mutations are the clustered average of individual MD trajectory outputs after 10 ns of molecular dynamics. Mutations on the left hand side (original sfGFP, H148C, H148T, H148N) together with H148S and original sfGFP have been experimentally analysed. For H148N, two model outcomes are shown: H148N[a] is from a starting point of the NH group of the carboxamide group closest to the chromophore phenol O; H148N[b] is from a starting of the O group of the carboxamide group closest to the chromophore phenol O. Polar interaction potential was assessed using the PyMOL[10] polar contacts tool, and if found shown as dashed yellow lines.

Distances between the backbone heavy atom [N] of the H-bond donor group [amide] and phenolate oxygen as shown as dashed orange lines. All distances are in nm.

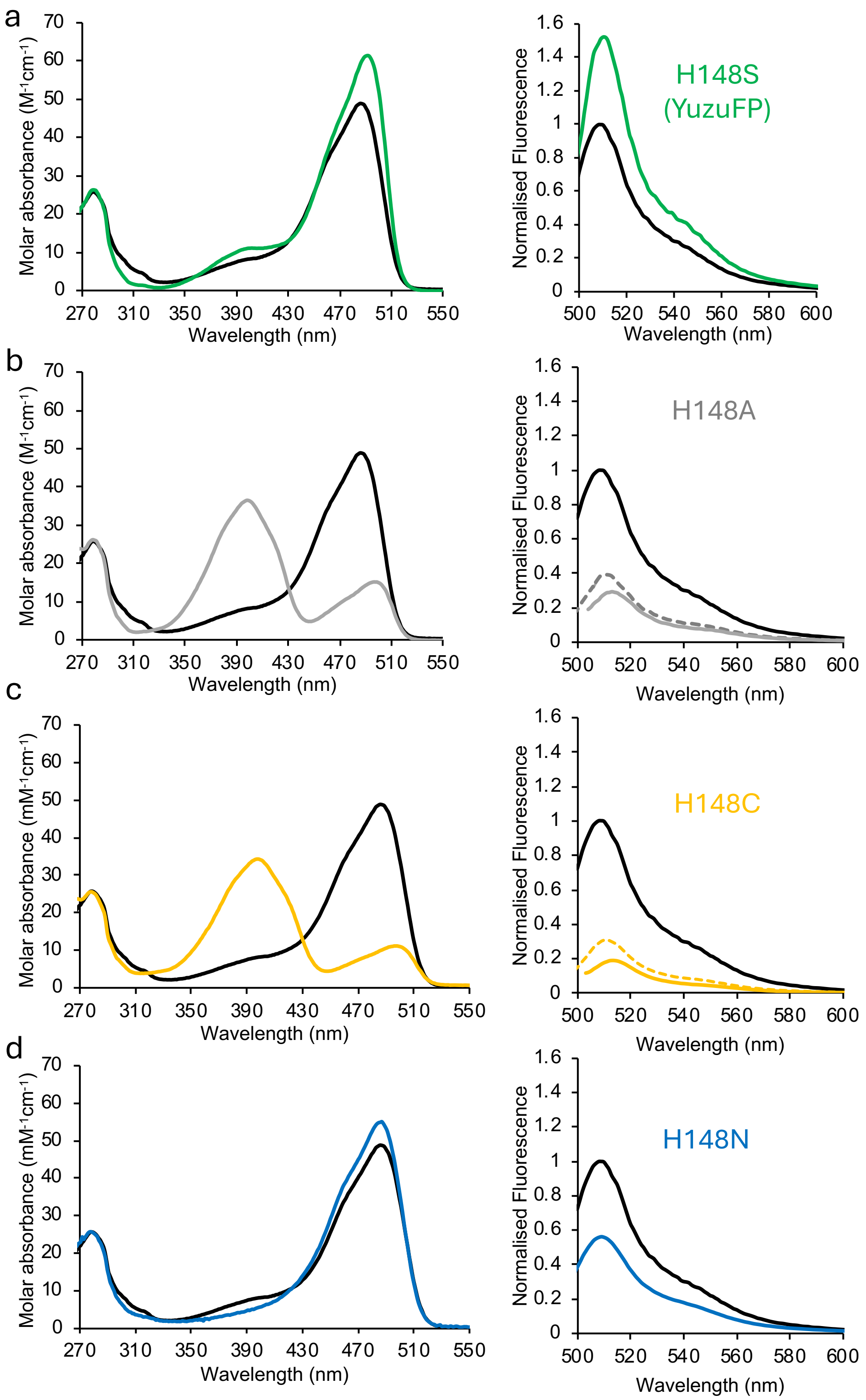

Supplementary Fig. 4. Absorbance (left panels) and Fluorescence emission spectra (right panels) for sfGFP (black in all cases) and the H148X variants experimentally tested. (a) H148S (YuzuFP; green), (b) H148A (grey), (c) H148C and (d) H148N. Fluorescence emission spectra

were normalised to wild-type sfGFP. For H148A, emission spectra were measured on excitation at either 400 nm (dashed line) or 497 nm (solid line), and for H148C on excitation at either 400 nm (dashed line) or 498 nm (solid line). Source data for plots in (a-d) are provided in Supplementary Data 2.

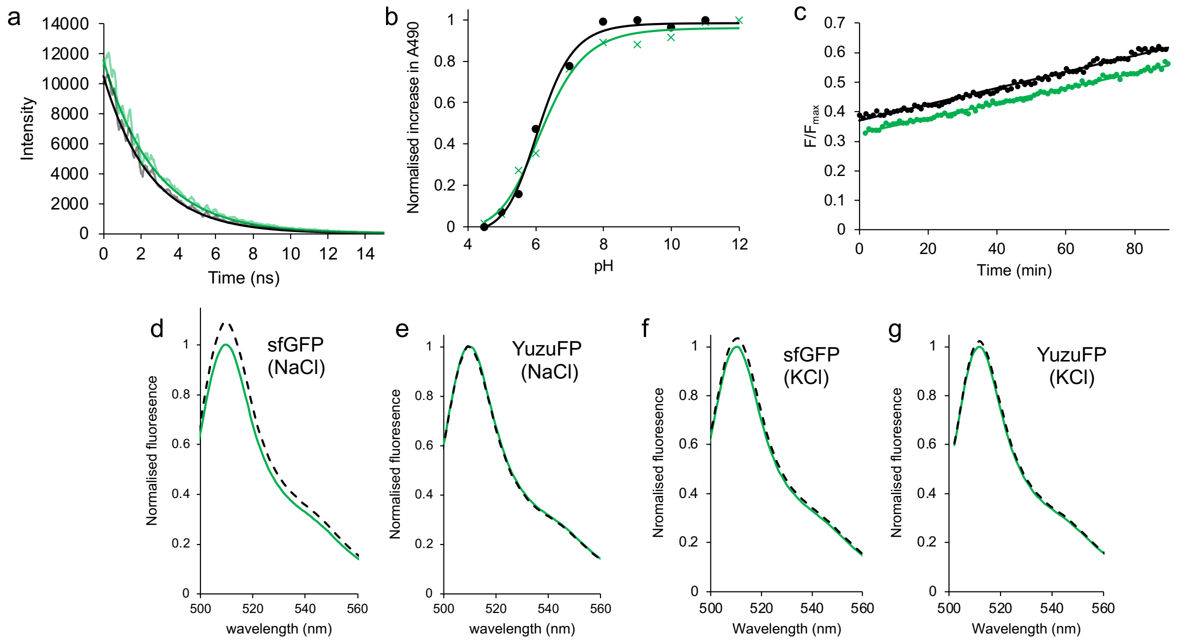

Supplementary Fig. 5. Analysis of YuzuFP. (a) The fluorescence intensity decay curve of YuzuFP (green) and sfGFP (black) on excitation at 473 nm. Data where fit to a single exponential decay in Graphpad Prism. (b) pKa of YuzuFP (green) and sfGFP (black) as measured by the increase in absorbance at 490 nm. Data where fit to a least squares fit sigmodal model in Graphpad Prism. (c) Maturation rate of sfGFP (black) and YuzuFP (green). Data where fit to a single exponential decay in Graphpad Prism, where F_{max} is the fluorescence emission after >10 hr post the rate measurements. (d-g) The effect of 150 mM salt (either NaCl or KCl) on fluorescence emission, as indicated in the figure. Green solid lines represent protein in 50 mM Tris pH 8.0 and dashed black lines in the presence of 150 mM salt. Source data for plots in (a-g) are provided in Supplementary Data 2.

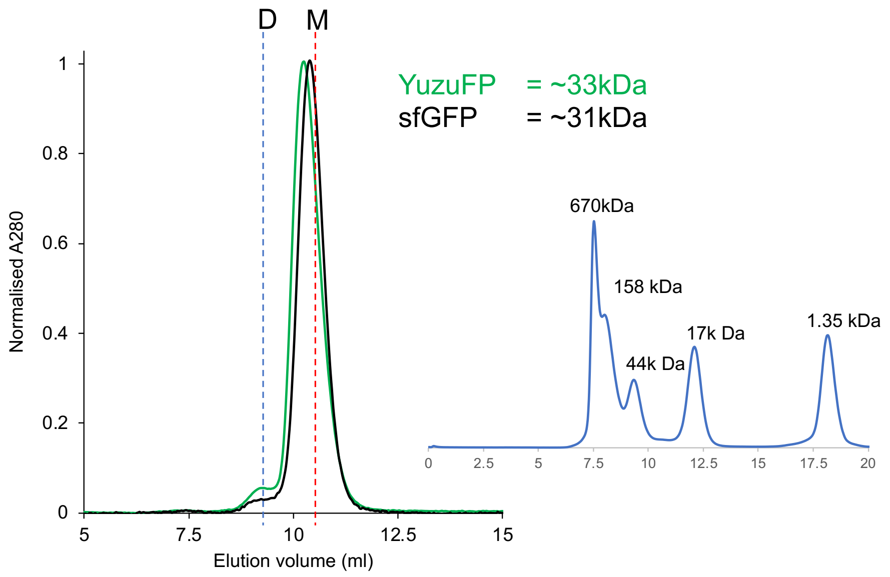

Supplementary Fig. 6. Analysis of quaternary structure YuzuFP by size exclusion chromatography (SEC). The black and green lines represent 50 μM sfGFP and 50 μM YuzuFP, respectively. SEC was performed using a calibrated Superdex 75 10/300GL column (inset). Estimated elution volumes for a theoretical monomer (M; 27 kDa) and dimer (D; 54 kDa) are shown by red and blue sashed lines, respectively. YuzuFP eluted very slightly earlier than sfGFP, giving an estimated molecular weight of ~33 kDa compared to 31 kDa for sfGFP. A small shoulder at ~9.3 mL suggests a small amount of dimer (~5%) may be present for both sfGFP and YuzuFP. Source data for plot are provided in Supplementary Data 2.

a.

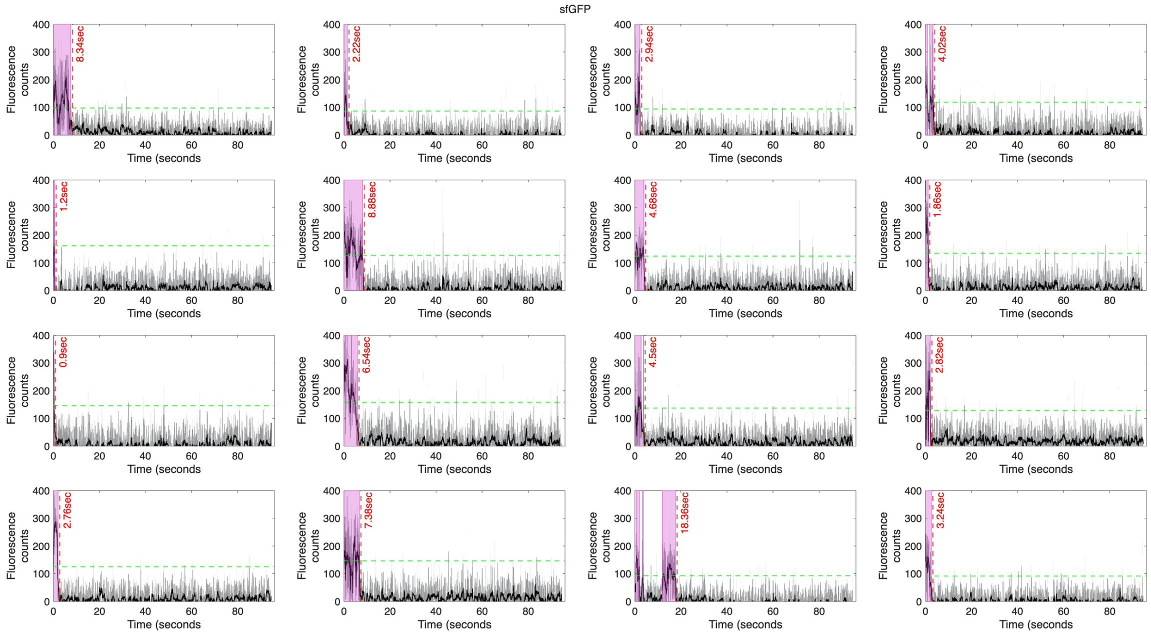

b.

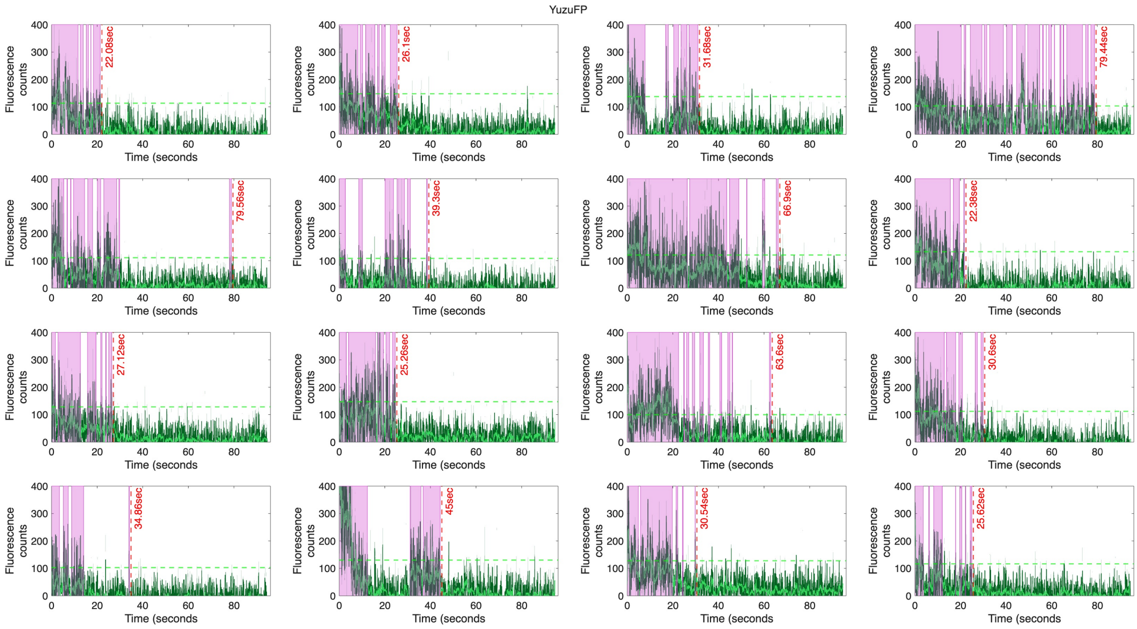

Supplementary Fig. 7. Exemplary single molecules fluorescence traces for (a) sfGFP and (b) YuzuFP . Single molecule data is extracted from a micrograph timeseries acquired using a total internal reflection fluorescence (TIRF) microscopy imaging system. Trace's are generated from the mean intensities of 4 by 4-pixel regions of interest corresponding to individual fluorophores. Raw data (grey and dark green) are plotted along side data passed through a forward-backward moving window average filter (black and light green). In order to clearly identify "on" and "off" fluorescence states thresholding of the raw data to 10 standard deviations beyond the mean background was used (green dashed line). This was combined with a temporal threshold of 0.9 seconds which was applied to remove intensity values that exceeded the intensity threshold but which were isolated in time by more than

0.9 seconds, in order to minimise false detections caused by background noise fluctuations. Those states deemed as being on according to these filtering parameters are also highlighted with purple shading. Using this in combination with a temporal threshold allowed for identification of photobleaching lifetimes of all single molecules (red dashed lines and times in seconds). Source data for plots in (a-b) are provided in Supplementary Data 2.

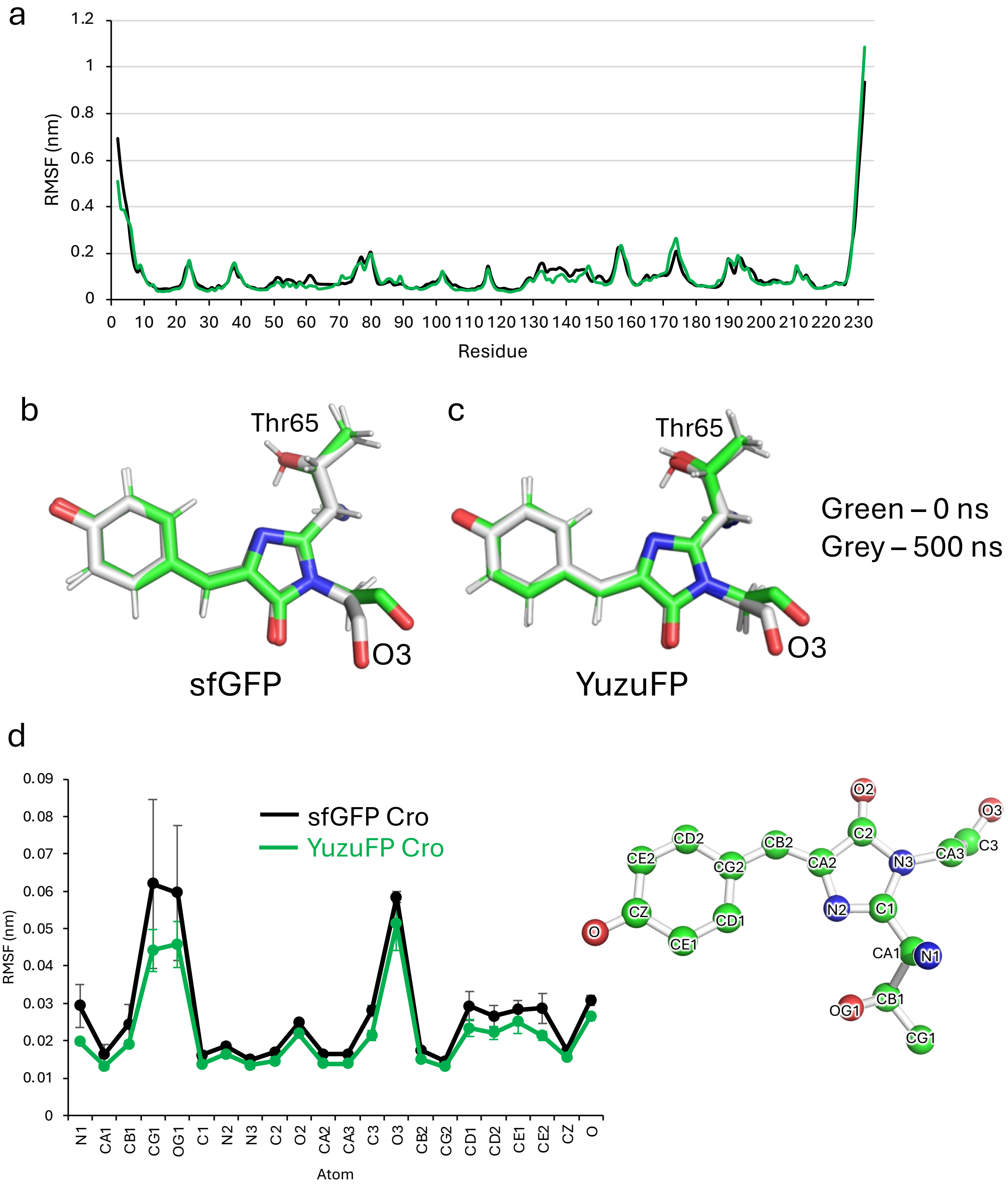

Supplementary Fig. 8. Change in backbone and chromophore structure over the course of MD simulation. (a) Per residue backbone (Cα) RMSF of sfGFP (black) and YuzuFP (green). RMSF values shown are an average of the 3 independent simulations. The chromophore structure at the start (green, 0 ns) and end (grey; 500 ns) of a single simulation for (b) sfGFP and (c) YuzuFP. (d) The root mean square fluctuation (RMSF) of each of the chromophore heavy atoms (as annotated in the molecular structure to the right) for sfGFP (black) and YuzuFP (green). The error bars represent the standard deviation between the 3 independent 500 ns simulations. Source data for plots in (a,d) are provided in Supplementary Data 2.

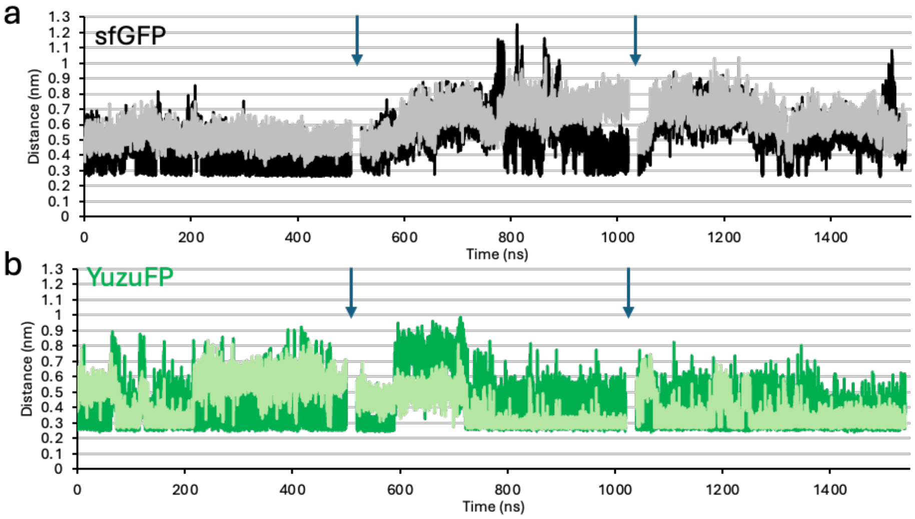

Supplementary Fig. 9. The pair-wise distance in (a) sfGFP and (b) YuzuFP, between chromophore phenol oxygen and residue 148 backbone and side chain H-bond donor heavy atoms. In (a), the backbone amide nitrogen is coloured grey and the side chain imidazole nitrogen of H148 is coloured black. In (b), the backbone amide nitrogen is coloured lime and the side chain hydroxyl oxygen of S148 is coloured green. The plots are a concatenation of the three 500 ns MD simulations, where the down arrows show the separation between each simulation. Source data for plots in (a-b) are provided in Supplementary Data 2.

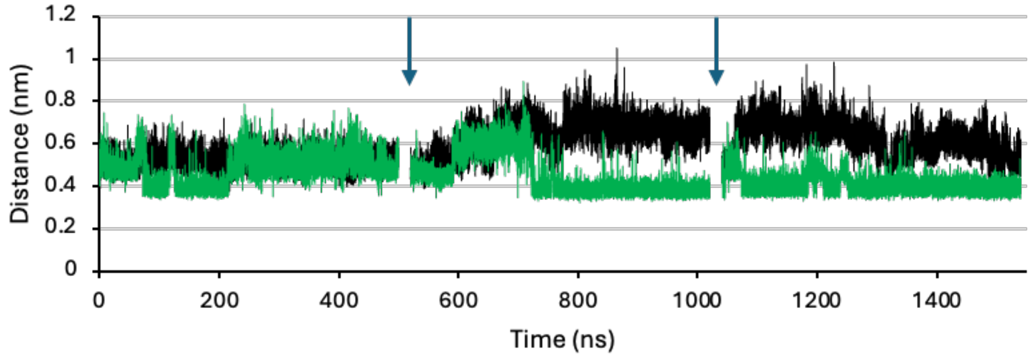

Supplementary Fig. 10. The distance between the chromophore phenol oxygen and the Ca of residue 148 in sfGFP (black) and YuzuFP (green). Each plot is a concatenation of 3 separate 500 ns simulations. The start and end of each simulation is shown by the down arrows. Source data for plots are provided in Supplementary Data 2.

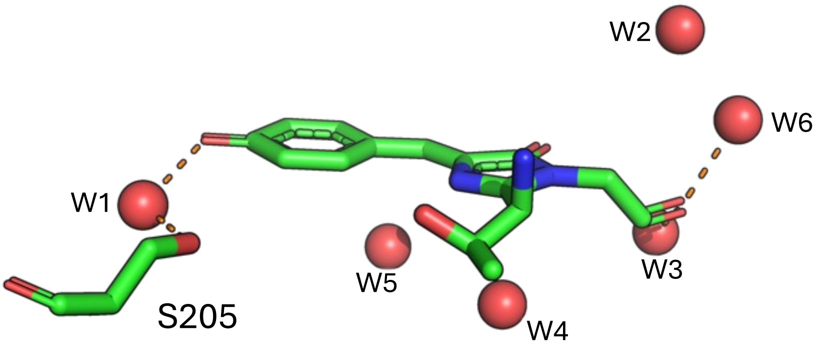

Supplementary Fig. 11. Water molecules within proximity of the chromophore. Red spheres are water molecules with the W1 water molecule labelled. Dashed lines are polar interactions between the chromophore and water molecules. The structure shown is PDB 2b3p.

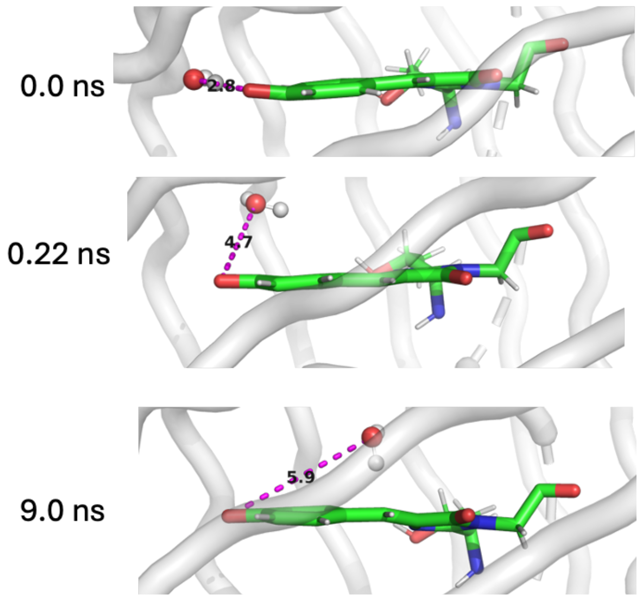

Supplementary Fig. 12. Position of water molecule W1 in sfGFP simulation 2. Within 0.22 ns, W1 is internalised and no longer able to form a H-bond with the chromophore phenol oxygen. The chromophore is coloured green and water molecule W1 shown as balled and stick. The dashed magenta lines and associated numeric value represent the distance between the chromophore oxygen and the W1 water molecule (in Ångstroms).

a sfGFP

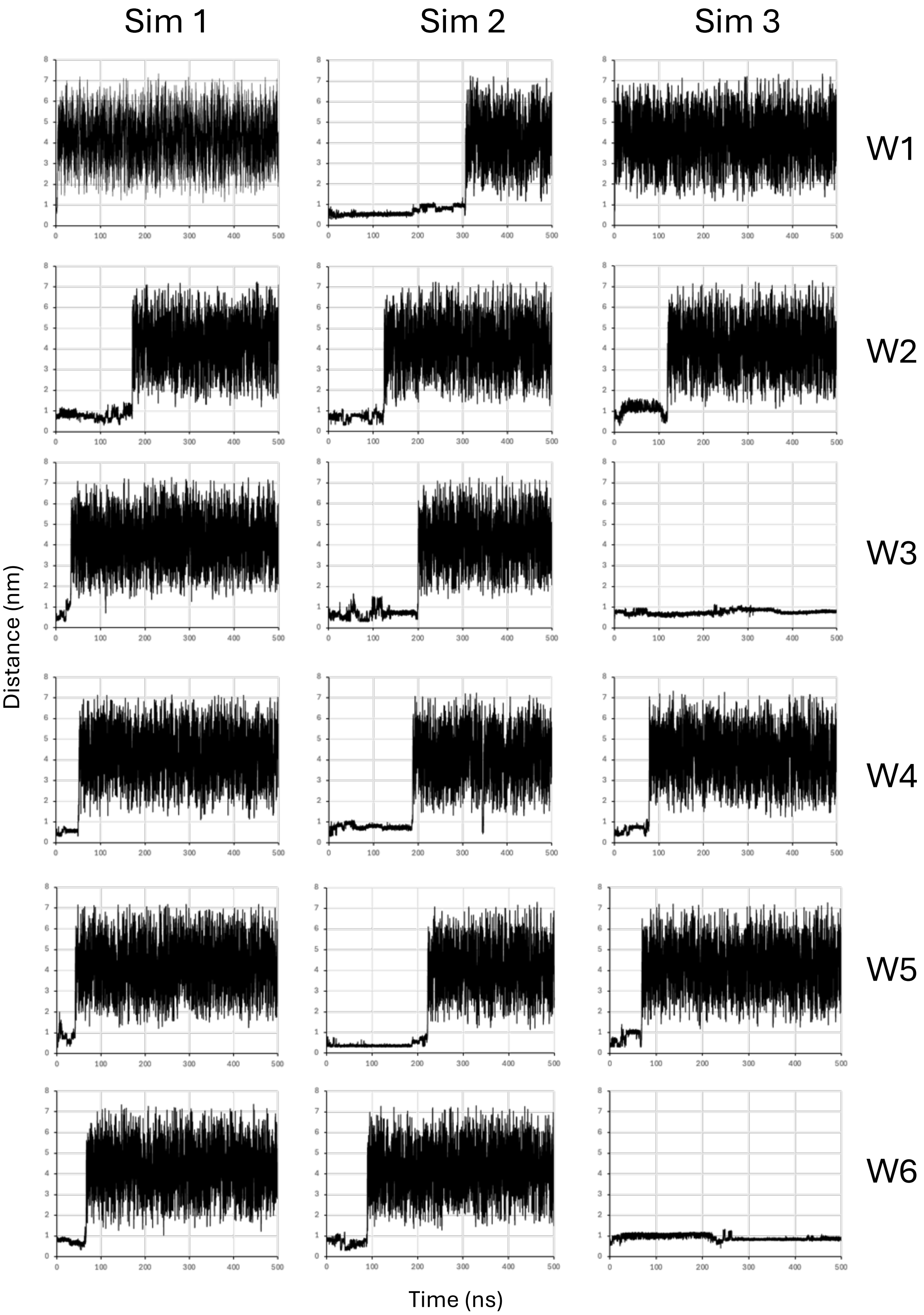

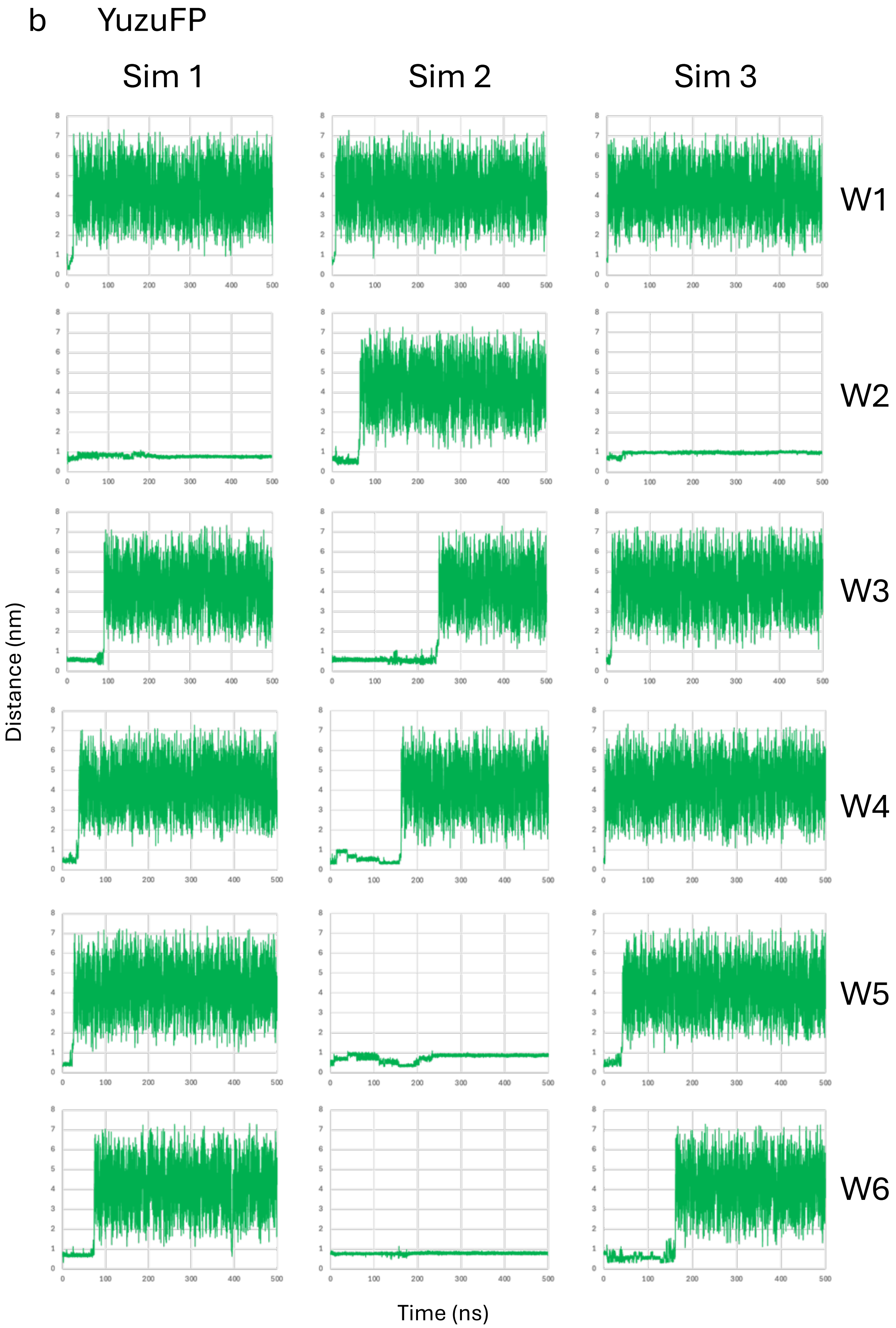
b
YuzuFP
Sim 1
Sim 2
Sim 3
W1
W2
W3
W4
W5
W6
Distance (nm)
Time (ns)

Supplementary Fig. 13. The pairwise distance between chromophore (atom 967 as part of the β-methylene bridge) and individual water molecule oxygen atoms (W1 to W6; see Supplementary Fig. 12 for their initial positions) from each 500 ns simulation (Sim 1 to 3) in (a) sfGFP and (b) YuzuFP. In sfGFP, W1 to W6 correspond to oxygen atom IDs 3642, 3618, 3624, 3663, 3708, 3609, respectively. For YuzuFP W1 to W6 correspond to oxygen atom IDs 3636, 3612, 3618, 3657, 3702, 3603. Source data for plots in (a-b) are provided in Supplementary Data 2.

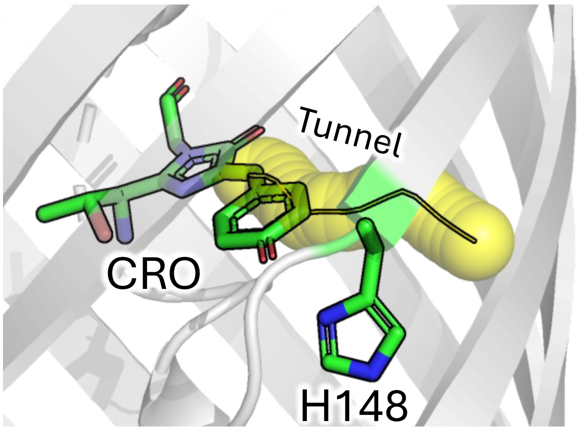

Supplementary Fig. 14. Tunnel (yellow; 1.1 Å probe radius) between CRO and solvent as calculated using CAVER [11,12] and the original PDB for sfGFP (2b3p)[9] .

Supplementary References

1. Reddington, S. C. *et al.* Different photochemical events of a genetically encoded phenyl azide define and modulate GFP fluorescence. *Angewandte Chemie - International Edition* **52**, 5974–5977 (2013).
2. Ando, R., Mizuno, H. & Miyawaki, A. Regulated fast nucleocytoplasmic shuttling observed by reversible protein highlighting. *Science* **306**, 1370–3 (2004).
3. Clavel, D. *et al.* Structural analysis of the bright monomeric yellow-green fluorescent protein mNeonGreen obtained by directed evolution. *Acta Crystallogr D Struct Biol* **72**, 1298–1307 (2016).
4. Ando, R. *et al.* StayGold variants for molecular fusion and membrane-targeting applications. *Nat Methods* **21**, 648–656 (2024).
5. Merzlyak, E. M. *et al.* Bright monomeric red fluorescent protein with an extended fluorescence lifetime. *Nat Methods* **4**, 555–557 (2007).
6. Subach, O. M. *et al.* Structural Characterization of Acylimine-Containing Blue and Red Chromophores in mTagBFP and TagRFP Fluorescent Proteins. *Chem Biol* **17**, 333–341 (2010).
7. Karasawa, S., Araki, T., Nagai, T., Mizuno, H. & Miyawaki, A. Cyan-emitting and orange-emitting fluorescent proteins as a donor/acceptor pair for fluorescence resonance energy transfer. *Biochemical Journal* **381**, 307–312 (2004).

8. Nienhaus, K., Nienhaus, G. U., Wiedenmann, J. & Nar, H. Structural basis for photo-induced protein cleavage and green-to-red conversion of fluorescent protein EosFP. *Proceedings of the National Academy of Sciences* **102**, 9156–9159 (2005).
9. Pédelacq, J. D., Cabantous, S., Tran, T., Terwilliger, T. C. & Waldo, G. S. Engineering and characterization of a superfolder green fluorescent protein. *Nat Biotechnol* **24**, 79–88 (2006).
10. Schrödinger, LLC. The PyMOL Molecular Graphics System. (2020).
11. Stourac, J. *et al.* Caver Web 1.0: identification of tunnels and channels in proteins and analysis of ligand transport. *Nucleic Acids Res* **47**, W414–W422 (2019).
12. Chovancova, E. *et al.* CAVER 3.0: A Tool for the Analysis of Transport Pathways in Dynamic Protein Structures. *PLoS Comput Biol* **8**, e1002708 (2012).
