# Supplementary material for: Molecular dynamics guided identification of a brighter variant of superfolder Green Fluorescent Protein with increased photobleaching resistance
Source: Commun Chem. 2025 Jun 5;8:174. doi: 10.1038/s42004-025-01573-4 (PMC12141695; doi:10.1038/s42004-025-01573-4)
Supplement: Supplementary file 2 — Description of Additional Supplementary Files [file 42004_2025_1573_MOESM2_ESM.pdf]

Description of Additional Supplementary Files

File name: Supplementary Data 1

Description: Source data from figures in the main manuscript.

File name: Supplementary Data 2

Description: Source Data from the Supplementary Information
